# Glioblastoma cellular MAP4K1 facilitates tumor growth and disrupts T effector cell infiltration

**DOI:** 10.26508/lsa.202301966

**Published:** 2023-09-21

**Authors:** Jin-Min Sun, Hong-Ye Fan, Yan Zhu, Ting-Ting Pan, Yong-Ping Wu, Dao-Yong Zhang, Xiao-Yu Hou

**Affiliations:** 1 Research Center for Biochemistry and Molecular Biology, Jiangsu Key Laboratory of Brain Disease Bioinformation, Xuzhou Medical University, Xuzhou, China; 2 State Key Laboratory of Natural Medicines, School of Life Science and Technology, China Pharmaceutical University , Nanjing, China; 3 Laboratory of Clinical and Experimental Pathology, Department of Pathology, Xuzhou Medical University, Xuzhou, China

## Abstract

MAP4K1 has been identified as a cancer immunotherapy target. Whether and how cancer cell-intrinsic MAP4K1 contributes to glioblastoma multiforme (GBM) progression remains unclear. We found that MAP4K1 was highly expressed in the glioma cells of human GBM specimens. High levels of *MAP4K1* mRNA were prevalent in *IDH*-WT and 1p/19q non-codeletion gliomas and correlated with poor prognosis of patients. MAP4K1 silencing inhibited GBM cell proliferation and glioma growth. Transcriptome analysis of GBM cells and patient samples showed that MAP4K1 modulated cytokine‒cytokine receptor interactions and chemokine signaling pathway, including *IL-18R* and *IL-6R*. Importantly, MAP4K1 loss down-regulated membrane-bound IL-18R/IL-6R by inhibiting the PI3K-AKT pathway, whereas MAP4K1 restoration rescued this phenotype and therefore GBM cell proliferation. MAP4K1 deficiency abolished GBM cell pro-proliferation responses to IL-18, suggesting an oncogenic role of MAP4K1 via the intrinsic IL-18/IL-18R pathway. In addition, GBM cell-derived MAP4K1 impaired T-cell migration and reduced CD8^+^ T-cell infiltration in mouse glioma models. Together, our findings provide novel insight into the pathological significance of GBM cell-intrinsic MAP4K1 in driving tumor growth and immune evasion by remodeling cytokine–chemokine networks.

## Abstract (precis)

MAP4K1 acts as a cancer cell-intrinsic driver of tumorigenesis and immune evasion in human gliomas, specifically glioblastoma multiforme, by modulating cytokine–chemokine signaling networks.

## Introduction

Gliomas are the most prevalent type of primary brain tumors in adults, and remain among the most difficult cancers to treat (Lapointe et al, 2018). Glioblastoma multiforme (GBM) is a highly malignant World Health Organization (WHO) grade IV glioma and accounts for 45.6% of primary malignant brain tumors (Wirsching et al, 2016). High aggression and immune evasion of GBM cause poor prognosis and death in patients, with a median survival time of only 12–15 mo (Osuka & Van Meir, 2017; Xu et al, 2020). A variety of cytokine and chemokine pathways are either associated with tumor growth and progression or shape the tumor immune microenvironment. A comprehensive understanding of GBM cell-intrinsic molecular regulatory events may provide promising targeted therapeutics for malignant gliomas.

MAP4K1, also known as hematopoietic progenitor kinase 1, belongs to the mammalian Ste20-like family of serine/threonine kinases. Physically, MAP4K1 is widely expressed during embryonic development and restricted to lymphohematopoietic organs in adults (Hu et al, 1996; Kiefer et al, 1996). In recent years, hematopoietic cell MAP4K1 has been established as an immunosuppressive regulator and implicated in the development of autoimmune diseases (Zhang et al, 2011) and several solid tumors (Si et al, 2020). Intracellular MAP4K1 negatively regulates T-cell- and dendritic cell-mediated immune responses, and loss of MAP4K1 or its kinase function confers enhanced antitumor efficiency to T cells and dendritic cells (Schulze-Luehrmann et al, 2002; Alzabin et al, 2009; Hernandez et al, 2018; Si et al, 2020). These studies suggest that a targeted drug of MAP4K1 may have potential for cancer immunotherapy. However, there are very few studies that assess the roles of non-hematopoietic cell MAP4K1 in tumor growth and progression.

Evidence indicated that tumorigenesis suppressor programmed cell death four decreases MAP4K1 expression to inhibit human colon carcinoma cell invasion (Yang et al, 2006; Wang et al, 2012). In human pancreatic cancer, MAP4K1 loss is associated with the development of invasive pancreatic ductal adenocarcinomas, and its restoration in pancreatic ductal adenocarcinoma cells inhibits cell proliferation (Wang et al, 2009) and reduces invasion potential by inducing degradation of oncogenic kinase AXL (Song et al, 2020). Given that cancer cell-intrinsic MAP4K1 may serve as a tumor promoter or suppressor, revealing its functional relevance in different tumor types is crucial.

In this study, we show that MAP4K1 is intrinsically expressed by cancer cells in human high-grade glioma (HGG) tissues. We evaluated the clinical relevance of MAP4K1 levels with prognosis and pathological features in patients with gliomas. Mechanistically, we noticed that GBM cell MAP4K1 remodels cytokine–chemokine networks in the tumor microenvironment. Our findings provide novel insights into the cellular and molecular mechanisms underlying glioma progression and targeted therapy for malignant gliomas.

## Results

### MAP4K1 proteins are distributed in glioma cells of HGG, in particular GBM (Grade IV)

**Figure 1.**
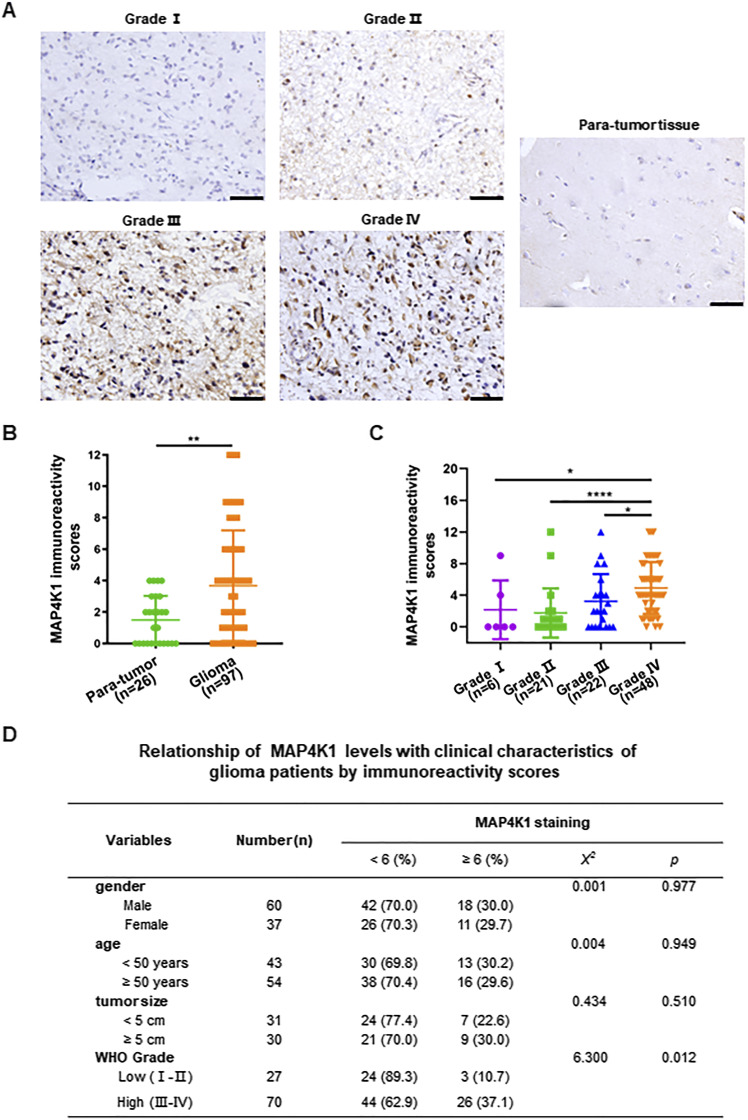
MAP4K1 expression is elevated in human high-grade gliomas. Human glioma (n = 97) and para-tumor tissues (n = 26) were stained with standard MAP4K1 immunohistochemistry (IHC). **(A)** Representative IHC images of MAP4K1 expression in human glioma tissues. Scale bar, 50 μm. **(B)** Quantification of MAP4K1 immunoreactivity scores in human para-tumor and glioma tissues. Data are represented as single data points and mean ± SD. **P < 0.01 (Mann‒Whitney *U* test). **(C)** Quantification of MAP4K1 immunoreactivity scores in grades I–IV of gliomas (n = 6, 21, 22, 48). Data are represented as single data points and mean ± SD. *P < 0.05, ****P < 0.0001 (one-way ANOVA). **(D)** Correlation analysis of pathological characteristics of glioma patients with MAP4K1 expression according to MAP4K1 IHC scores. Data were analyzed by the Mann–Whitney *U* test and *χ*^*2*^ test.

First, we explored whether MAP4K1 proteins are expressed by cancer cells in human glioma samples. Immunohistochemistry (IHC) analysis of tissue microarray slides showed that MAP4K1 proteins exhibited higher expression levels in glioma tissues than in para-tumor tissues and were mainly localized in the cytoplasm of glioma cells (Fig 1A and B). Furthermore, we found that the elevated expression of MAP4K1 was associated with the clinicopathological characteristics of glioma patients (Fig 1C and D). The levels of MAP4K1 were positively correlated with glioma WHO grade but not gender, age or tumor size. MAP4K1-overexpressing glioma cells were primarily present in HGG (grades III–IV), especially in GBM (Grade IV), indicating a positive correlation of MAP4K1 levels with glioma progression.

### MAP4K1 is primarily expressed in isocitrate dehydrogenase gene (*IDH*)-WT and 1p/19q noncodeletion gliomas and correlated with poor prognosis of patients

**Figure 2.**
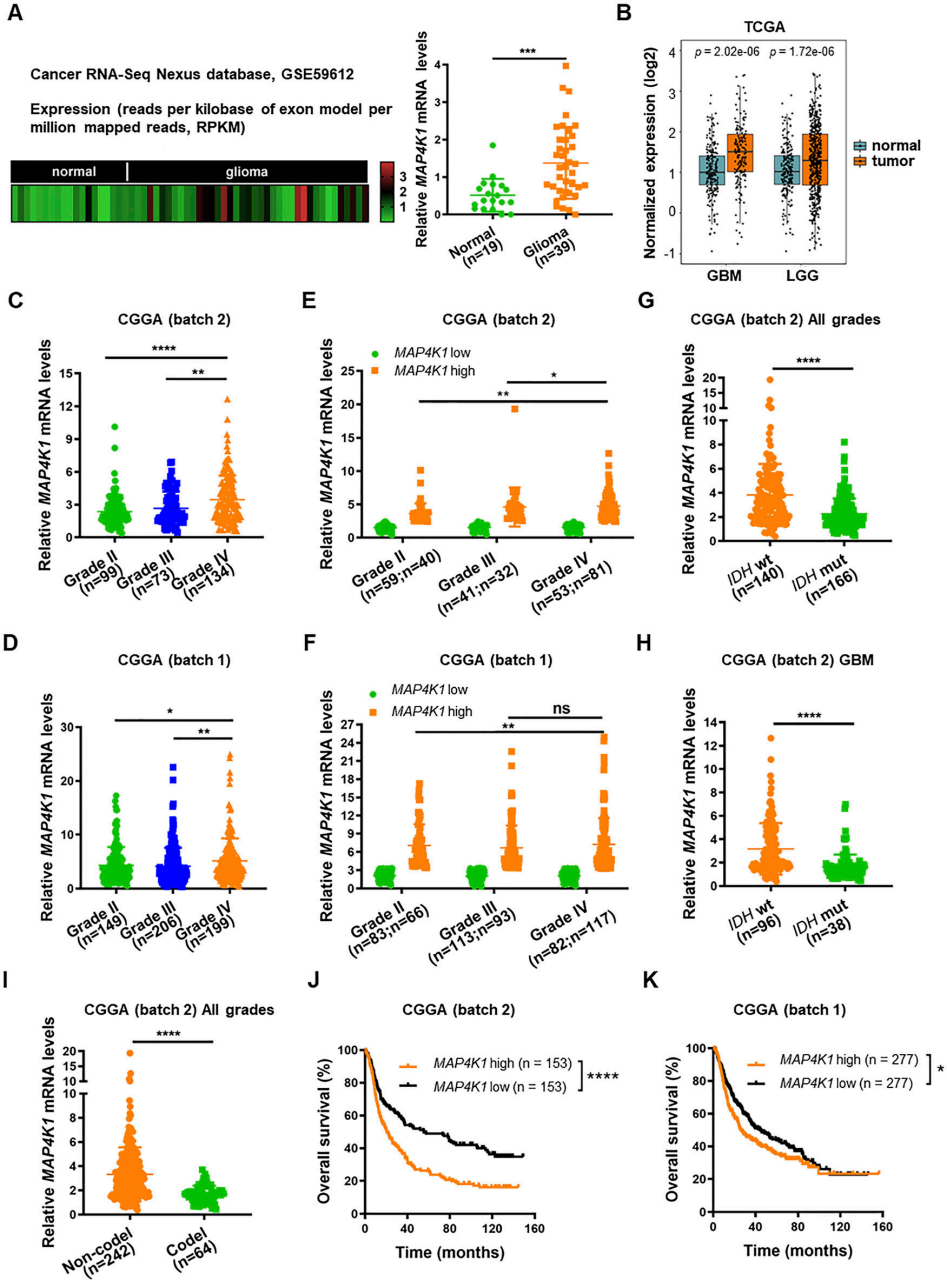
High MAP4K1 levels are correlated with poor prognosis in glioma patients. **(A)** Heatmap (left) and statistical analysis (right) of *MAP4K1* mRNA expression in human normal brain (n = 19) and glioma tissues (n = 39). The data were collected from the Cancer RNA-Seq Nexus platform (GSE59612). Data are represented as single data points and mean ± SD. ***P < 0.001 (*t* test). **(B)** Analysis of *MAP4K1* mRNA levels in normal cerebral cortex samples (n = 207), glioblastoma multiforme (n = 166), and low-grade gliomas (n = 523). The data were from The Cancer Genome Atlas. Data are represented as single data points and mean ± SD (*t* test). **(C, D)** *MAP4K1* mRNA expression in different pathological grades (II–IV). The data were from batch 2 ((C), n = 306) and batch 1 ((D), n = 554) of the Chinese Glioma Genome Atlas (CGGA). Data are represented as single data points and mean ± SD. *P < 0.05, **P < 0.01, ****P < 0.0001 (one-way ANOVA). **(E, F)** Cases of high and low *MAP4K1* mRNA levels in different pathological grades (II–IV). The data were from CGGA batch 2 ((E), grade II, low n = 59, high n = 40; grade III, low n = 41, high n = 32; grade IV, low n = 53, high n = 81) and batch 1 ((F), grade II, low n = 83, high n = 66; grade III, low n = 113, high n = 93; grade IV, low n = 82, high n = 117). Data are represented as single data points and mean ± SD. ns, no significance, *P < 0.05, **P < 0.01 (*χ*^*2*^ test). **(G, H, I)** Analysis of *MAP4K1* mRNA levels according to isocitrate dehydrogenase gene (*IDH*) phenotypes, WT or mutation (mut), in all grades of gliomas ((G), n = 306) and glioblastoma multiforme ((H), n = 134), and 1p/19q status (codeletion or non-codeletion) in all grades of gliomas ((I), n = 306). The data were from CGGA (batch 2). Data are represented as single data points and mean ± SD. ****P < 0.0001 (*t* test). **(J, K)** Overall survival analysis of *MAP4K1*-low and -high groups of glioma patients. The data were from CGGA batch 2 ((J), n = 306) and batch 1 ((K), n = 554). *P < 0.05, ****P < 0.0001 (Kaplan‒Meier method, log-rank test). **(E, F, J, K)** The samples were categorized into *MAP4K1*-low and -high groups using median expression levels of *MAP4K1* mRNA as the cutoff value in (E, F, J, K).

Next, the analysis of mRNA sequencing data from the Cancer RNA-Seq Nexus database (GSE59612) showed elevated *MAP4K1* mRNA levels in human gliomas compared with those in normal brain tissues (Fig 2A), which was consistent with the IHC results. We further confirmed this finding by analyzing The Cancer Genome Atlas (TCGA) data (Fig 2B) and Chinese Glioma Genome Atlas (CGGA) data (Fig 2C and D). TCGA data analysis showed that *MAP4K1* mRNA was overexpressed in both low-grade gliomas (n = 523) and GBM (n = 166) compared with that in normal cerebral cortex samples (n = 207) (Fig 2B). Moreover, CGGA data (batch 2 and batch 1) showed that the levels of *MAP4K1* mRNA in GBM were higher than those in low-grade gliomas (Fig 2C and D), and the percentage of high *MAP4K1* mRNA level cases increased with the grades of gliomas (Fig 2E and F), supporting a positive correlation of MAP4K1 levels with a malignant phenotype of gliomas.

*IDH* (*IDH1* and *IDH2*) genotype and 1p/19q status are important molecular biomarkers in the clinical diagnosis and prognosis evaluation of gliomas. MAP4K1 was differentially expressed in gliomas according to *IDH* status, WT or mutation (mut) (Fig 2G and H). *MAP4K1* mRNA presented higher levels in *IDH* wt gliomas than in the *IDH* mut subset (Fig 2G) and was especially prevalent in *IDH* wt GBM (Fig 2H). Similarly, *MAP4K1* mRNA was predominantly prevalent in the 1p/19q non-codeletion glioma cohort (Fig 2I). Considering that 1p/19q codeletion or *IDH* mutation predicts longer overall survival for patients with diffuse gliomas, we further analyzed the data from the CGGA database to evaluate the prognostic value of MAP4K1 (Fig 2J and K). We found that patients with comparatively higher levels of *MAP4K1* mRNA had a lower rate of overall survival than patients with low levels of *MAP4K1* mRNA, suggesting that MAP4K1 up-regulation signifies a poorer prognosis for patients with gliomas.

### GBM cell MAP4K1 promotes cell proliferation and regulates cell survival and the cell cycle

**Figure S1.**
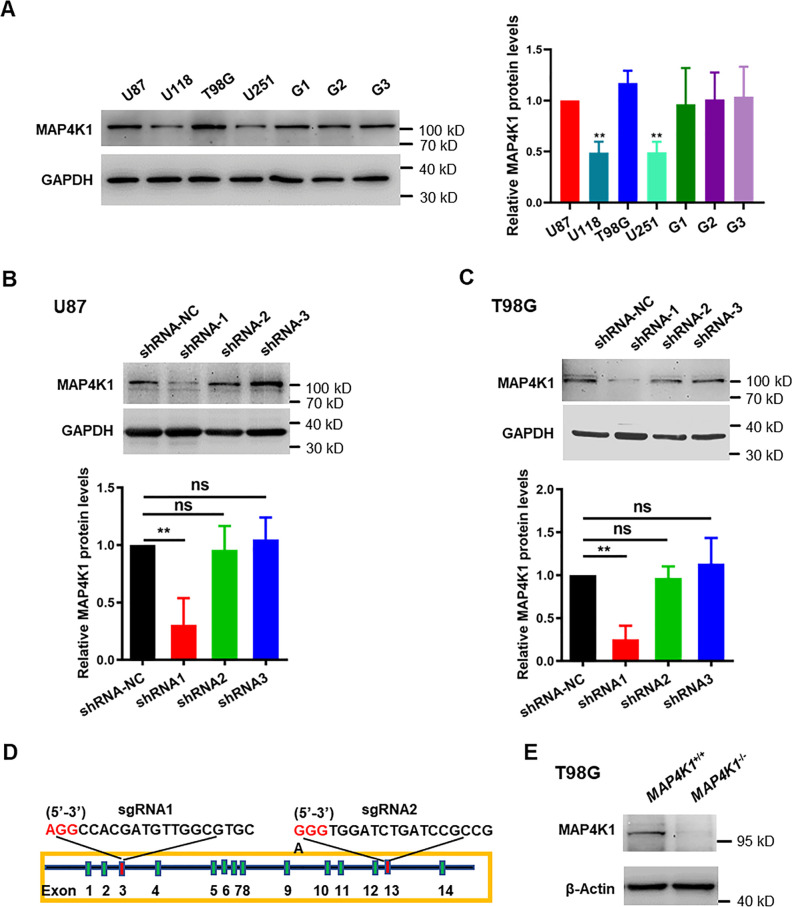
MAP4K1 proteins are relatively highly expressed in glioblastoma multiforme (GBM) U87 and T98G cell lines and patient-derived GBM cells. **(A)** MAP4K1 protein expression was detected in different GBM cell lines (U87, U118, T98G, U251) and primary patient-derived GBM cells (G1, G2, G3) by immunoblotting. The relative levels of MAP4K1 were normalized to that in U87 group. **(B, C)** Representative figures of immunoblot analysis and statistical results of three shRNA knockdown efficiencies in U87 and T98G cells. **(A, B, C)** Data are represented as the mean ± SD (n = 3). ns, no significance, **P < 0.01 (one-way ANOVA). **(D, E)** MAP4K1 was knocked out by the CRISPR/Cas9 system in T98G cells. **(D)** Schematic of specific gRNAs designed to target exon 3 and exon 13 of the *MAP4K1* gene. **(E)** Immunoblot analysis of MAP4K1 protein expression in MAP4K1 knockout T98G cells (n = 3). Source data are available for this figure.

**Figure 3.**
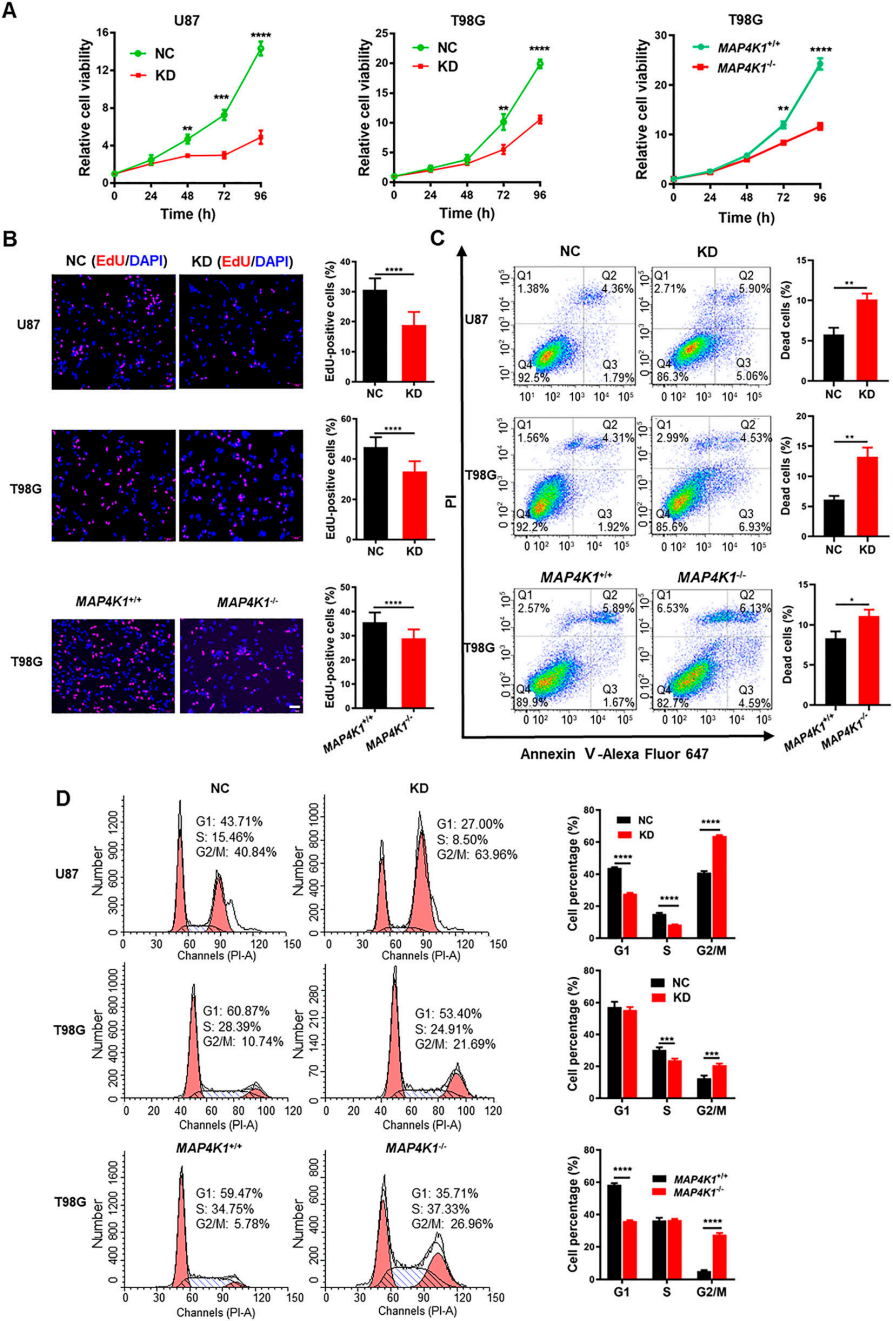
MAP4K1 promotes the proliferation and inhibits the death of glioblastoma multiforme cells. **(A)** CCK8 assay of cellular viability at different times (24, 48, 72, and 96 h) in MAP4K1 knockdown (KD) or knockout (*MAP4K1*^−/−^) U87 and T98G cells and their control groups (NC, *MAP4K1*^*+/+*^), respectively (n = 15, the data were from three independent experiments). **(B)** 5-ethynyl-2-deoxyuridine assay of cell proliferation in MAP4K1-KD U87 and T98G or *MAP4K1*^−/−^ T98G cells and their NC groups 48 h after seeding (n = 20, the data were from three independent experiments). Scale bar, 100 μm. **(C)** Flow cytometry analysis of the rates of dead cells, which indicates Annexin V- plus PI-positive cells 48 h after seeding (n = 3, the data were from an independent experiment, and the same experiment was repeated three times). **(D)** Cell cycle analysis by flow cytometry showed G2/M arrest in MAP4K1-KD or knockout glioblastoma multiforme cells 72 h after seeding (n = 3, the data were from an independent experiment, and the same experiment was repeated three times). Data are represented as the mean ± SD. *P < 0.05, **P < 0.01, ***P < 0.001, ****P < 0.0001. **(A, B, C, D)** *t* tests (B, C) and two-way ANOVA (A, D).

To evaluate the involvement of MAP4K1 in human GBM growth, we detected the proliferation and death of GBM cells upon gene silencing or genetic ablation of MAP4K1. In contrast to GBM cell lines U118 and U251, MAP4K1 proteins were relatively highly expressed in U87 and T98G cell lines and primary patient-derived GBM cells (Fig S1A). Immunoblot assays confirmed the efficiency of MAP4K1 knockdown (KD) by specific shRNAs in U87 and T98G cells (Fig S1B and C) and MAP4K1 knockout (*MAP4K1*^−^/^−^) by the CRISPR/cas9 system in T98G cells (Fig S1D and E). The Cell Counting Kit-8 (CCK-8) assay showed a significant reduction in cell proliferation in MAP4K1-KD U87 and T98G cells compared with that in the respective negative control (NC) groups (Fig 3A). The down-regulation of proliferation was further confirmed in *MAP4K1*^−/−^ T98G cells in comparison with that in the *MAP4K1*^+/+^ group (Fig 3A). The proliferation rate of U87 and T98G GBM cells was assessed by labeling cells with 5-ethynyl-2-deoxyuridine (EdU). The percentage of EdU-positive proliferating cells decreased significantly in the MAP4K1-KD and knockout groups (Fig 3B).

**Figure S2.**
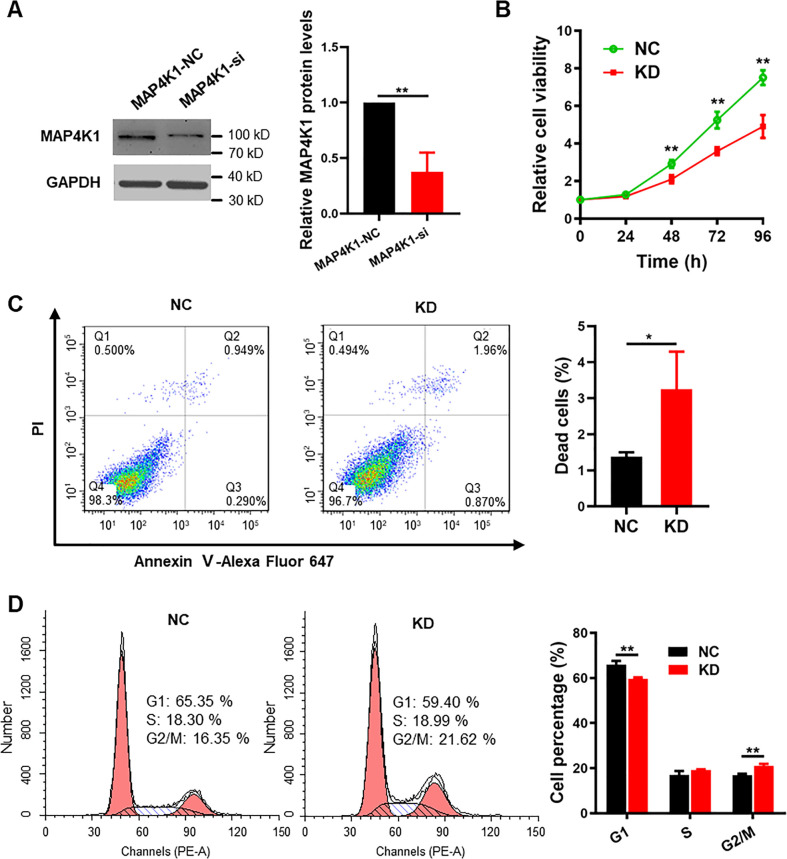
MAP4K1 promotes proliferation and inhibits apoptosis in patient-derived glioblastoma G1 cells. **(A)** Representative images of immunoblot analysis and statistical results of MAP4K1 siRNA knockdown efficiency in G1 cells. Data are represented as the mean ± SD (n = 3, *t* test), **P < 0.01. **(B)** Cellular viability detected by CCK8 (24, 48, 72, and 96 h) in G1 cells transduced with either a nontargeting control siRNA (NC) or MAP4K1-targeting siRNA (KD). Data are represented as the mean ± SD (n = 15, the data were from three independent experiments), **P < 0.01 (two-way ANOVA). **(C, D)** Cell death after 48 h of seeding (C) and cell cycle distribution after 72 h of seeding (D) were analyzed by flow cytometry in MAP4K1-KD and control G1 cells. **(C, D)** Data are represented as the mean ± SD (n = 3, the data were from an independent experiment, and this experiment was repeated three times), *P < 0.05 (*t* test in (C)), **P < 0.01 (two-way ANOVA in (D)). Source data are available for this figure.

Flow cytometry (FCM) was used to determine the proportion of dead cells and the distribution of cell cycle phases. We found that MAP4K1 deficiency in U87 and T98G cells resulted in an increased incidence of cell death, which indicates Annexin V- plus PI-positive cells (Fig 3C). In addition, MAP4K1-KD or knockout caused the cell cycle to arrest during the G2/M phase in both U87 and T98G cells (Fig 3D). Similarly, in G1 primary patient-derived GBM cells, MAP4K1 silencing also inhibited cell proliferation (Fig S2A and B) and induced cell death and G2/M phase arrest (Fig S2C and D).

Thus, MAP4K1 is associated with cell proliferation, survival, and cell cycle regulation of GBM cells, suggesting that GBM cell MAP4K1 plays a critical role in tumor growth and progression.

### GBM cell MAP4K1 promotes tumor growth and cell proliferation in mouse and human gliomas

**Figure 4.**
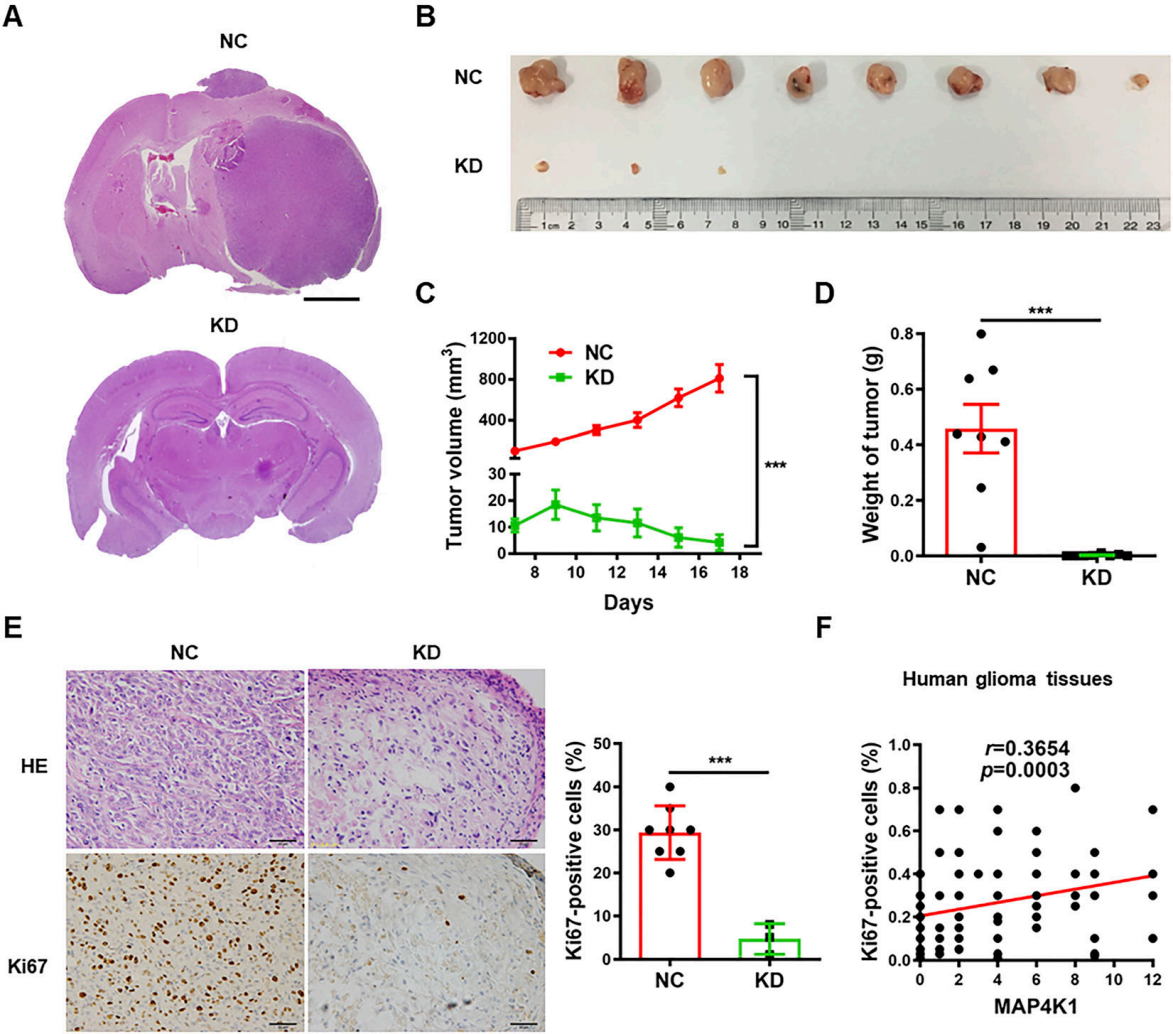
MAP4K1 of glioblastoma multiforme cells promotes glioma growth in vivo. **(A)** Representative hematoxylin and eosin (HE) staining images of mouse intracranial gliomas. Stable MAP4K1-knockdown (KD) U87 cells or negative control (NC) cells were orthotopically implanted into athymic nude mice (n = 4). Scale bar, 0.8 mm. **(B, C, D, E)** Gross images of tumors (B), tumor growth curve (C), tumor weight (D), and HE staining/Ki67 immunohistochemistry staining (E) in mouse subcutaneous gliomas. Scale bar, 50 μm. Stable MAP4K1-KD U87 cells or NC cells were subcutaneously implanted into athymic nude mice (n = 8). **(C, D)** Data are represented as mean ± SEM (two-way ANOVA in (C) and Mann‒Whitney *U* test in (D)). **(E)** Data are represented as single data points and mean ± SD (*t* tests in (E)) ***P < 0.001. **(F)** Correlation analysis of MAP4K1 immunoreactivity scores with proliferation rates (% Ki67-positive cells) in human glioma tissues (n = 97). Correlation analysis was performed by Spearman correlation.

To further confirm the impact of MAP4K1 on glioma growth and progression, stable MAP4K1-KD U87 cells were orthotopically and subcutaneously implanted into athymic nude mice. MAP4K1-KD in GBM cells led to marked growth suppression of intracranial (Fig 4A) and subcutaneous tumors (Fig 4B–D) in athymic nude mice. The volume and weight of subcutaneous tumors were dramatically decreased (Fig 4C and D). IHC staining with proliferation marker Ki-67 showed that fewer cells were Ki-67-positive in MAP4K1-KD tumors relative to NC tumors (Fig 4E), indicating that MAP4K1 promotes glioma cell proliferation in vivo. The result was consistent with the correlation analysis of MAP4K1 immunoreactivity scores (derived from the staining in Fig 1) with the Ki67-positive rate in human glioma samples (Fig 4F), further confirming an oncogenic role of MAP4K1 in gliomas.

### Transcriptome profiles reveal orchestration of cytokine–chemokine signaling networks by MAP4K1 in GBM

**Figure 5.**
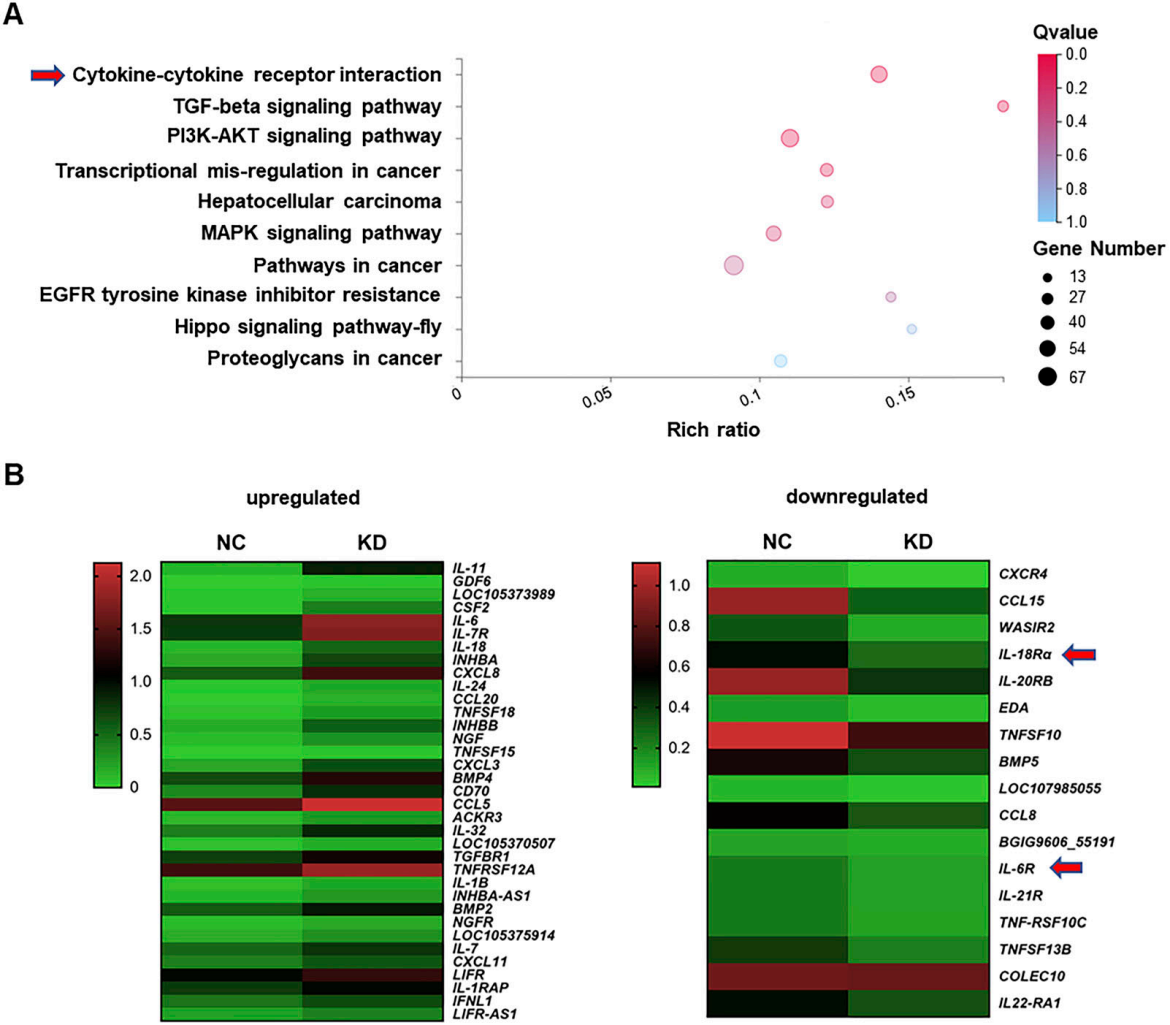
MAP4K1 silencing alters transcriptomic profiling of glioblastoma multiforme cells. **(A)** Kyoto Encyclopedia of Genes and Genome pathway enrichment analysis of transcriptome data based on differentially expressed genes in MAP4K1-knockdown (KD) T98G cells and their negative control (NC). Cytokine‒cytokine receptor interaction and PI3K-AKT signaling were among the top three enriched pathways. **(B)** Heatmap of differentially expressed genes in the cytokine‒cytokine receptor pathways (left, up-regulated genes; right, down-regulated genes. *IL-18R* and *IL-6R* are down-regulated with MAP4K1-KD in T98G cells.

To investigate the molecular mechanisms by which MAP4K1 promotes tumorigenesis in gliomas, we assessed transcriptomic changes after MAP4K1 down-regulation in T98G cells using next-generation RNA sequencing (RNA-seq). Kyoto Encyclopedia of Genes and Genome (KEGG) pathway enrichment analysis of the RNA-seq data identified that cytokine‒cytokine receptor interactions and the PI3K-AKT signaling pathway were involved in the top three hits (Fig 5A). Differentially expressed genes (DEGs) in the cytokine‒cytokine receptor pathway showed markedly down-regulated mRNA levels of cytokines and chemokines, including *IL-18R* and *IL-6R*, in MAP4K1-KD T98G cells compared with those in the NC group (Fig 5B).

**Figure 6.**
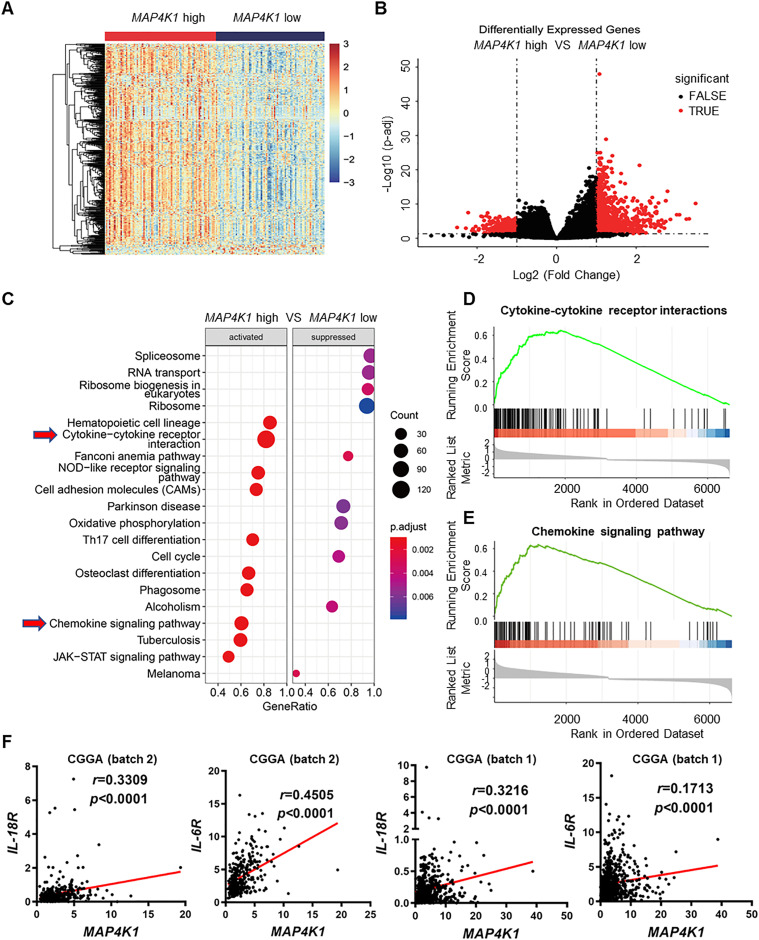
MAP4K1 is correlated with cytokine‒cytokine receptor interactions and chemokine signaling pathways in human gliomas. **(A, B)** Heatmap (A) and volcano plot (B) show differentially expressed genes in *MAP4K1*-high and -low human glioblastoma multiforme. The data were from The Cancer Genome Atlas-glioblastoma multiforme dataset (n = 166) and categorized into high- and low-expression groups using median expression levels of *MAP4K1* mRNA as the cutoff value. **(C, D, E)** Gene Set Enrichment Analysis pathway enrichment analysis based on differentially expressed genes in *MAP4K1*-high and -low groups (C). **(D)** MAP4K1 is positively correlated with cytokine–cytokine receptor interactions. *P* = 0.000103, NES = 2.872095, *Q*-value = 0.000550. **(E)** MAP4K1 is positively correlated with chemokine signaling pathways. *P* = 0.000106, NES = 2.707719, *Q*-value = 0.000551. **(F)** Correlation analysis of *MAP4K1* mRNA levels with *IL-18R* and *IL-6R* levels. The data were from batch 2 and batch 1 of the Chinese Glioma Genome Atlas (batch 2, n = 325, batch 1, n = 693, *r* and *P*-values are indicated).

Furthermore, we analyzed the RNA-seq data from the TCGA-GBM dataset. GBM samples were categorized into *MAP4K1*-high and *MAP4K1*-low groups using median expression levels of *MAP4K1* mRNA as the cutoff value. DEseq2 was used to identify the DEGs between the two groups. Many more up-regulated genes were found in *MAP4K1*-high samples than in *MAP4K1*-low samples (Fig 6A and B). Gene set enrichment analysis identified various signaling pathways, including cytokine‒cytokine receptor interactions and chemokine signaling pathway, with significantly elevated expression levels in the *MAP4K1*-high group (Fig 6C–E), which was consistent with the transcriptomic profiling of T98G cells. A variety of cytokines are involved in tumor growth and progression. CGGA data analysis revealed that high levels of *IL-18* or *IL-6* mRNA predict a poor prognosis for glioma patients (data not shown). More importantly, cytokine receptors on the GBM cell surface determine the intrinsic biological responses of GBM cells to cytokines. Correlation analysis from different CGGA datasets (batch 2 and batch 1) showed a positive correlation of *MAP4K1* with either *IL-18R* or *IL-6R* mRNA levels in human gliomas (Fig 6F).

These results imply that MAP4K1 dictates intrinsic cytokine–chemokine pathways and therefore glioma growth and progression.

### MAP4K1 up-regulates IL-18R/IL-6R levels to facilitate GBM cell proliferation

**Figure 7.**
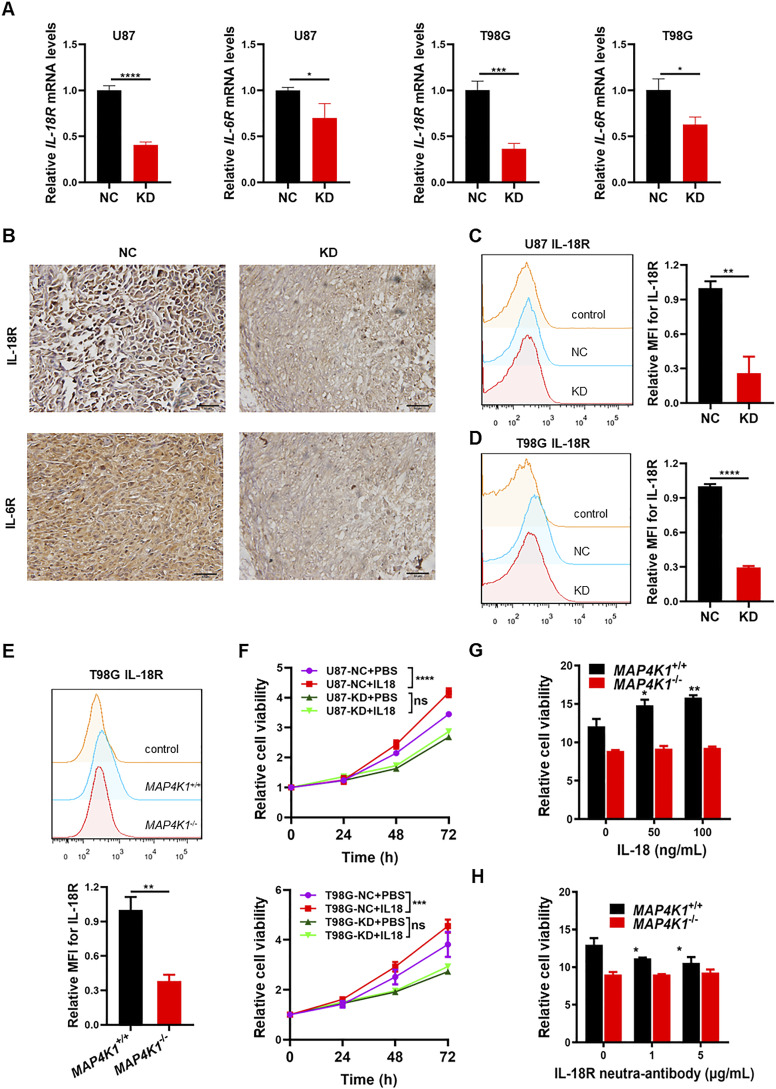
MAP4K1 enhances the expression of IL-18R and IL-6R and promotes the proliferation of glioblastoma multiforme cells. **(A)** Real-time quantitative PCR analyses of *IL-18R* and *IL-6R* mRNA levels in MAP4K1-knockdown (KD) U87 and T98G cells and their negative control (NC) cells (n = 9, the data were from three independent experiments). **(B)** IL-18R and IL-6R protein expression detected by immunohistochemistry in mouse glioma tissues constructed by MAP4K1-KD (n = 3) and NC (n = 8) of U87 cells. Scale bar, 50 μm. **(C, D, E)** Respective flow cytometry histograms and relative levels of the mean fluorescence intensity for membrane-bound IL-18R in MAP4K1-KD or knockout (*MAP4K1*^−/−^) U87, T98G, and respective control cells 48 h after seeding (n = 3, the data were from an independent experiment, and the same experiment was repeated three times). **(F, G, H)** CCK8 assay of cell viability. The data were from three independent experiments. **(F)** MAP4K1-KD and NC cells were stimulated with IL-18 (100 ng/ml) and detected at different time points (24, 48, and 72 h) (n = 11). **(G)** *MAP4K1*^−/−^ and *MAP4K1*^+/+^ T98G cells were treated with different doses of IL-18 (0, 50, and 100 ng/ml). **(H)** IL-18R was blocked with a neutralizing antibody (0, 1, 5 μg/ml). **(G, H)** Cell viability was determined at 96 h (n = 15 in (G, H)). In (A, C, D, E, F, G, H), data are presented as the mean ± SD. In (A, C, D, E), the data were analyzed by *t* tests. In (F, G, H), data were analyzed by two-way ANOVA. ns, not significant, *P < 0.05, **P < 0.01, ***P < 0.001, ****P < 0.0001.

**Figure S3.**
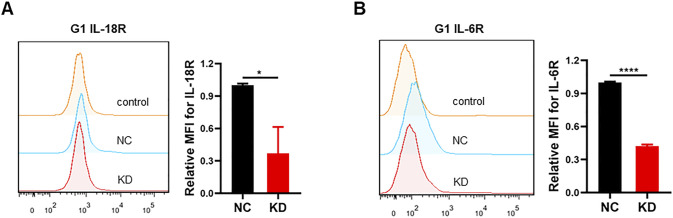
Membrane-bound IL-18R and IL-6R are down-regulated in MAP4K1-knockdown (KD) G1 cells. **(A, B)** Respective flow cytometry (histograms and relative levels of the mean fluorescence intensity of membrane-bound IL-18R (A) and IL-6R (B) after 48 h of seeding in MAP4K1-knockdown (KD) G1 cells and control cells (NC). Data are represented as the mean ± SD (n = 3, the data were from an independent experiment, and this experiment was repeated three times), *P < 0.05, ****P < 0.0001 (*t* test).

**Figure S4.**
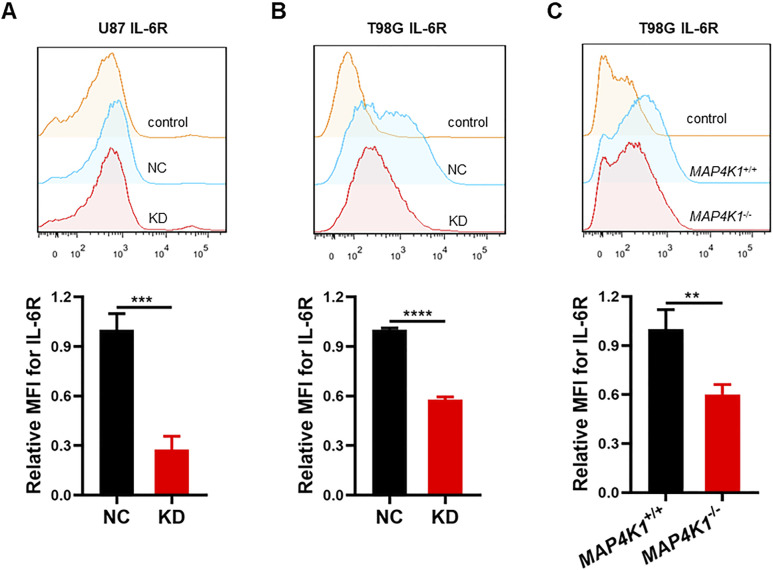
Membrane-bound IL-6R is down-regulated with MAP4K1 silencing in glioblastoma multiforme cells. **(A, B, C)** Respective flow cytometry histograms and relative levels of the mean fluorescence intensity of membrane-bound IL-6R after 48 h of seeding in MAP4K1-knockdown (KD) U87 cells (A), T98G cells (B), and MAP4K1 knockout (*MAP4K1*^*−/−*^) T98G cells (C) and their control cells. Data are represented as the mean ± SD (n = 3, the data were from an independent experiment, and this experiment was repeated three times), **P < 0.01, ***P < 0.001, ****P < 0.0001 (*t* test).

Does MAP4K1 regulate the IL-18R and IL-6R signaling pathways in various GBM cells and in vivo? We found that MAP4K1-KD obviously decreased the levels of *IL-18R* and *IL-6R* mRNA in U87 and T98G cells (Fig 7A). IHC staining showed that both IL-18R and IL-6R protein levels were down-regulated by MAP4K1-KD in mouse subcutaneous glioma tissues constructed with U87 cells compared with those in NC groups (Fig 7B). More importantly, in primary patient-derived G1 cells, MAP4K1-KD reduced the levels of membrane-bound IL-18R and IL-6R (Fig S3A and B). We confirmed the reduction of membrane-bound IL-18R (Fig 7C–E) and IL-6R (Fig S4A–C) in MAP4K1-deficient U87 and T98G cells compared with those in the respective controls (NC). Previous studies have indicated that IL-6 promotes glioma cell proliferation (Jiang et al, 2017; Liu et al, 2021). Here, we sought to identify the pro-proliferation responses of GBM cells to IL-18 stimulation. IL-18 stimulation promoted the proliferation of U87 and T98G cells, which was abolished by MAP4K1-KD (Fig 7F). This result was confirmed by MAP4K1 ablation in T98G cells (Fig 7G). The proliferation of *MAP4K1*^+/+^ T98G cells was inhibited after IL-18R was blocked by a neutralizing antibody, whereas no obvious change was found in *MAP4K1*^−/−^ T98G cells (Fig 7H). Thus, MAP4K1 plays an oncogenic role, at least partially, via the intrinsic IL-18/IL-18R signaling pathway.

**Figure 8.**
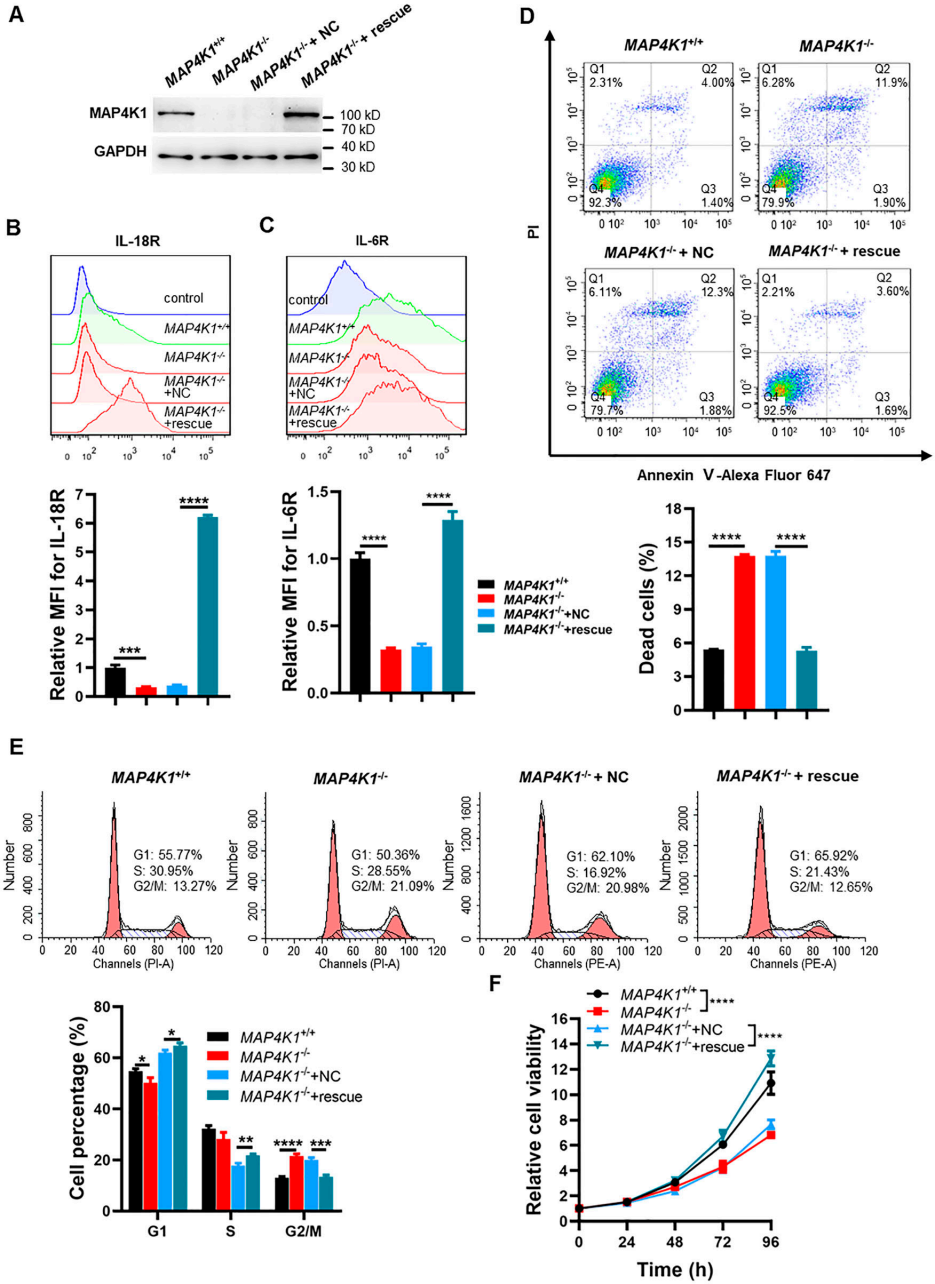
MAP4K1 restoration rescues the membrane expression of IL-18R and IL-6R and glioblastoma multiforme cell proliferation. **(A)** MAP4K1 expression was recovered in *MAP4K1*^−/−^ T98G cells, as confirmed by immunoblotting (n = 3). **(B, C)** Respective flow cytometry histograms and relative levels of the mean fluorescence intensity of membrane-bound IL-18R (B) and IL-6R (C) in MAP4K1 rescue *MAP4K1*^−/−^ T98G cells and *MAP4K1*^+/+^ T98G cells. Data are represented as the mean ± SD (n = 3, the data were from an independent experiment, and the same experiment was repeated three times). **(D, E)** Cell death ((D), 48 h after seeding) and cell cycle distribution ((E), 72 h after seeding) were analyzed by flow cytometry in MAP4K1 rescue T98G cells. Data are represented as the mean ± SD (n = 3, the data were from an independent experiment, and the same experiment was repeated three times). **(F)** Cell viability detection by CCK8 at different times (24, 48, 72, and 96 h) in MAP4K1 rescue T98G cells. Data are represented as the mean ± SD (n = 15, the data were from three independent experiments). *P < 0.05, **P < 0.01, ***P < 0.001, ****P < 0.0001 (two-way ANOVA). Source data are available for this figure.

The restoration of MAP4K1 expression in *MAP4K1*^−/−^ T98G cells further confirmed the impact of MAP4K1 on IL-18R/IL-6R membrane expression and GBM cell proliferation. The levels of membrane-bound IL-18R and IL-6R were recovered by MAP4K1 restoration (Fig 8A–C). Meanwhile, cell death (Fig 8D), the cell cycle (Fig 8E), and cell proliferation (Fig 8F) were also rescued after MAP4K1 restoration.

**Figure S5.**
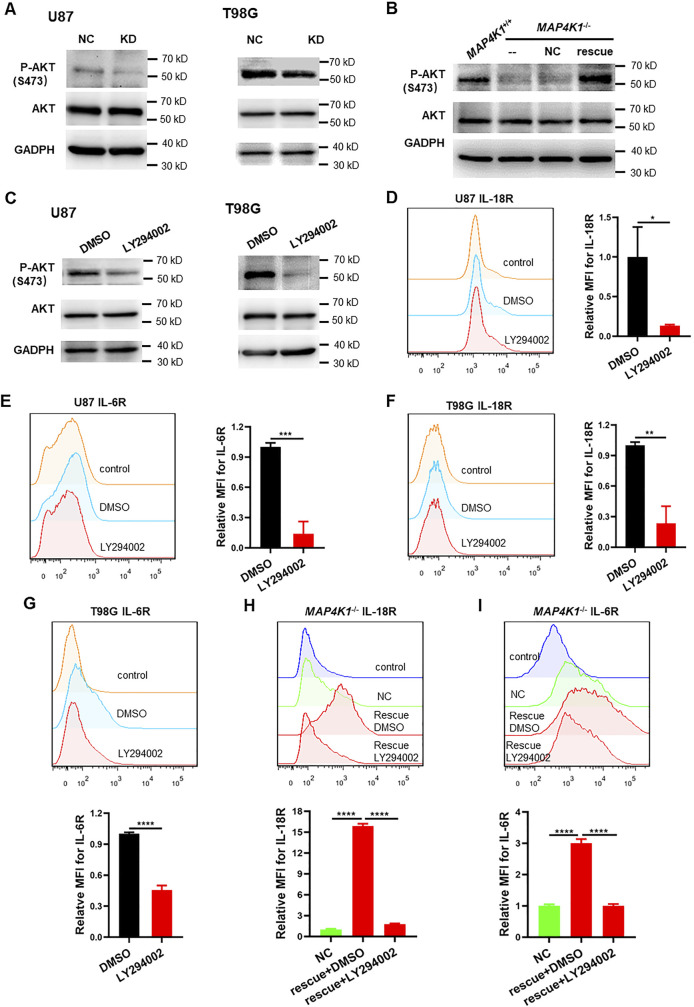
MAP4K1 regulates IL-18R and IL-6R expression by the PI3K-AKT pathway. **(A)** Representative immunoblot graphs of P-AKT (S473) and AKT levels in MAP4K1-knockdown (KD) U87 and T98G cells and control cells (NC) (n = 3). **(B)** Representative immunoblot graphs of P-AKT (S473) and AKT levels in MAP4K1 rescue T98G cells and control cells (NC) (n = 3). **(C)** Representative immunoblot graphs of P-AKT (S473) and AKT levels after treatment with the PI3K inhibitor LY294002 (25 μM) or vehicle DMSO for 48 h in U87 and T98G cells (n = 3). **(D, E, F, G)** Respective flow cytometry histograms and relative levels of the mean fluorescence intensity of membrane-bound IL-18R and IL-6R after treatment with the PI3K inhibitor LY294002 (25 μM) or vehicle DMSO for 72 h in U87 and T98G cells. **(H, I)** Respective flow cytometry histograms and relative levels of the mean fluorescence intensity of membrane-bound IL-18R (H) and IL-6R (I) after treatment with LY294002 (25 μM) or vehicle DMSO for 72 h in MAP4K1 rescue T98G cells. **(D, E, F, G, H, I)** Data are represented as the mean ± SD (n = 3, the data were from an independent experiment, and this experiment was repeated three times), *P < 0.05, **P < 0.01, ***P < 0.001, ****P < 0.0001 (*t* test in (D, E, F, G), one-way ANOVA in (H, I)). Source data are available for this figure.

To test how MAP4K1 regulates the IL-18R and IL-6R signaling pathways in GBM cells, we examined the phosphorylation (activation) of AKT on serine 473 (P-AKT) and found that P-AKT was blocked after MAP4K1-KD (Fig S5A), whereas MAP4K1 restoration rescued P-AKT levels (Fig S5B). The selective PI3K inhibitor LY294002 reduced P-AKT levels (Fig S5C) and IL-18R/IL-6R membrane expression in U87 and T98G cell lines (Fig S5D–G). MAP4K1 restoration failed to induce IL-18R and IL-6R expression when treatment with LY294002 (Fig S5H and I). Therefore, MAP4K1 up-regulates IL-18R and IL-6R expression through the PI3K-AKT-signaling pathway.

These results indicate that MAP4K1 facilitates GBM cell proliferation and tumor growth through intrinsic IL-18/IL-18R and IL-6/IL-6R signaling pathways.

### MAP4K1 knockdown increases CD8^+^ T-cell infiltration in mouse gliomas

**Figure 9.**
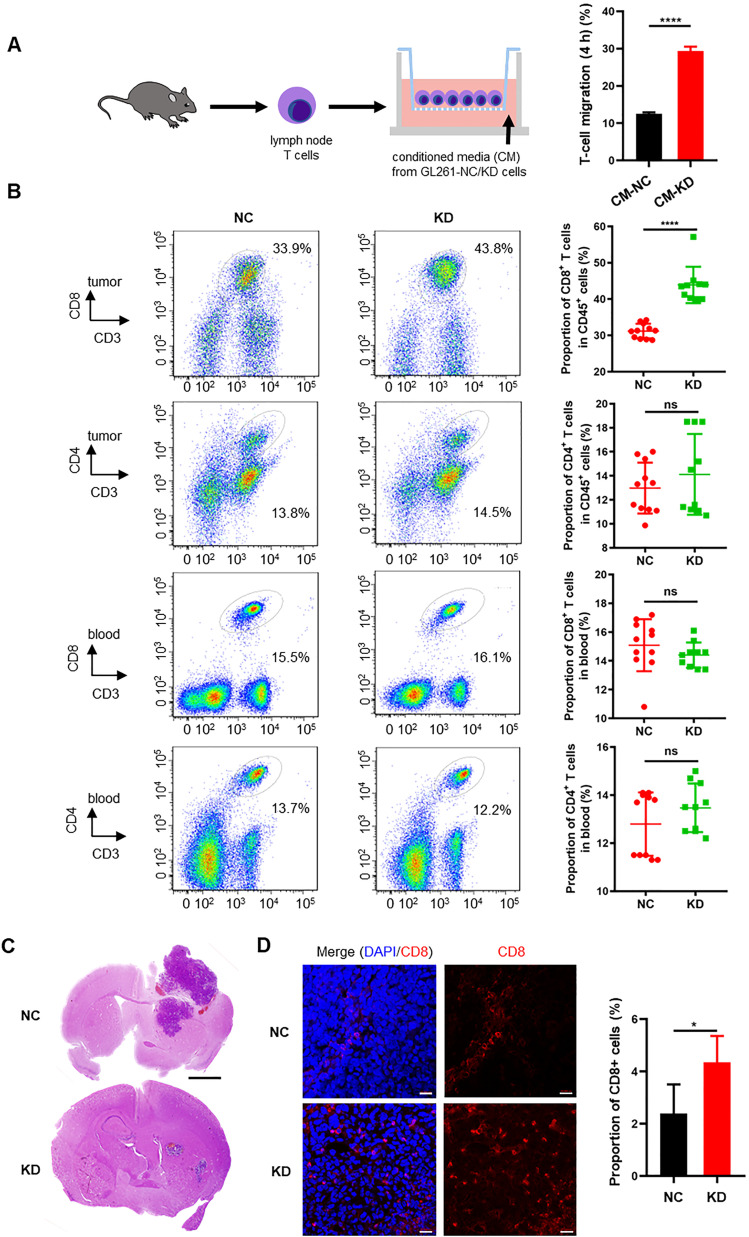
MAP4K1 down-regulation increases the infiltration of CD8^+^ T cells in tumor tissues of subcutaneous and intracranial glioma models. **(A)** The migration of T cells was detected by chemotaxis experiments 4 h after treatment with conditioned medium (CM) from MAP4K1-knockdown (KD) and negative control (NC) GL261 cells. Data are represented as the mean ± SD (n = 3, the data were from an independent experiment, and this experiment was repeated three times). ****P < 0.0001 (*t* test). **(B)** Flow cytometry analysis of CD8^+^ and CD4^+^ T-cell proportions of CD45^+^ cells in the tumor tissues and peripheral blood of mouse subcutaneous glioma models constructed with MAP4K1-KD GL261 cells (n = 10) and NC cells (n = 11). Data are represented as the mean ± SD. ns, no significance, ****P < 0.0001 (*t* test). **(C)** Representative hematoxylin and eosin (HE) staining images of tumors from the intracranial glioma models constructed with MAP4K1-KD GL261 and NC cells (n = 4). Scale bar, 0.8 mm. **(D)** Proportions of CD8^+^ T-cell infiltration were measured by immunofluorescence in intracranial gliomas constructed with MAP4K1-KD GL261 and NC cells (n = 4). Scale bar, 20 μm. Data are represented as the mean ± SD. *P < 0.05 (*t* test).

**Figure S6.**
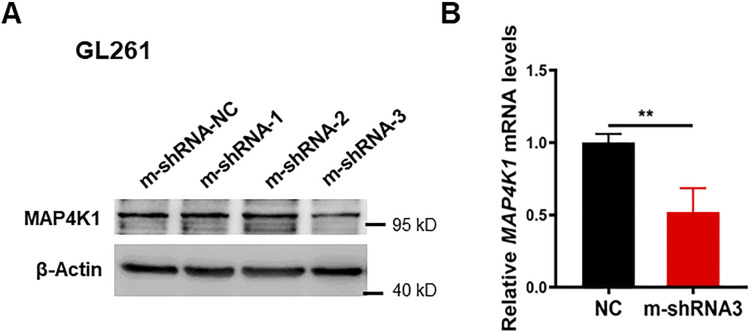
Immunoblot and quantitative PCR analysis of MAP4K1 knockdown efficiency in GL261 cells. **(A, B)** MAP4K1 knockdown efficiency was detected by immunoblotting ((A), n = 3) or quantitative PCR ((B), n = 9, the data are shown from three independent experiments, *t* test, **P < 0.01) in GL261 cells transduced with three mouse MAP4K1 shRNAs or their negative control (shRNA-NC). Source data are available for this figure.

Gliomas accompanied by a degree of tumor-infiltrating lymphocytes (TILs), especially CD8^+^ T cells, are a predictive factor for favorable outcomes. Based on the transcriptome profiles of human GBM (Figs 5 and 6), we hypothesized that GBM cell-derived MAP4K1 modulates the expression and secretion of various cytokines and chemokines to retrain the immune responses in the microenvironment of malignant gliomas. To test the above hypothesis, we performed chemotaxis experiments to examine the effects of the cultured supernatant of the mouse GBM cell line GL261 after MAP4K1-KD on T-cell migration in vitro (Figs 9A and S6A and B). As shown in Fig 9A, the conditioned medium of MAP4K1-KD GL261 cells enhanced the migration of T cells compared with that of NC cells, suggesting that GBM cells autonomously restrain TIL accumulation in an intrinsic MAP4K1-dependent manner. Correlatedly, in subcutaneous mouse GL261 gliomas, the MAP4K1-KD group exhibited increased intratumoral infiltration of CD8^+^ T cells, rather than CD4^+^ T cells, compared with that of the NC groups (Fig 9B). The proportion of peripheral CD8^+^ or CD4^+^ T cells did not show any differences between the KD and NC groups in subcutaneous glioma models (Fig 9B). Furthermore, MAP4K1 KD in GL261 cells limited tumor growth in mouse intracranial glioma models (Fig 9C). Immunofluorescence analysis showed an obviously increased accumulation of CD8^+^ T cells in intracranial MAP4K1-KD-GL261 gliomas (Fig 9D). These results suggest that cell-autonomous MAP4K1 by GBM cells negatively regulates TIL recruitment to tumors.

## Discussion

The standard treatment for patients with HGG is maximal surgical resection followed by radiotherapy with concurrent chemotherapy or adjuvant chemotherapy. The development of molecule-targeted therapy and immunotherapy is expected to improve patients’ clinical outcomes (Hoang-Minh & Mitchell, 2018; Pellerino et al, 2018). Previous studies found that MAP4K1 in T cells and dendritic cells limits antitumor responses in several solid tumors (Schulze-Luehrmann et al, 2002; Alzabin et al, 2009; Si et al, 2020). MAP4K1 expression is not restrictively confined to lymphohematopoietic cells. Here, our findings suggest that MAP4K1 is expressed in the cytoplasm of human glioma cells and that its levels are positively correlated with the histopathological grading of gliomas. MAP4K1 facilitates GBM cell proliferation, survival, and tumor growth by remodeling cytokine–chemokine signaling networks, including the IL-18R and IL-6R pathways. On the other hand, GBM cell-intrinsic MAP4K1 inhibits CD8^+^ TIL invasion and migration to promote immune evasion in malignant gliomas. The present data contribute to a better understanding of the pathological relevance of MAP4K1 and the underlying mechanisms in GBM progression, indicating that MAP4K1 is a potential molecular therapeutic target for malignant gliomas.

We found that high levels of MAP4K1 are associated with a malignant phenotype and subclasses (*IDH*-wt, 1p/19q noncodeletion) of human gliomas, signifying poor prognosis of patients. In GBM, *IDH* mut and 1p/19q codeletion subsets present with low levels of *MAP4K1* mRNA, implying that mutated *IDH*, particularly codeleted 1p/19q, may constrain MAP4K1 expression. To date, patients with *IDH* wt gliomas exhibit the poorest outcomes, and few targeted agents are therapeutically effective for this cohort. All 1p/19q-codeleted gliomas harbor *IDH* mutations, which is accompanied by improved survival in patients (Eckel-Passow et al, 2015). The link between *IDH* mutations and favorable prognosis in patients with gliomas remains unknown. Our data provide a novel mechanism by which the down-regulated MAP4K1 signaling pathway is responsible for the improved survival of patients with *IDH* mut gliomas. Recently, reduced cytolytic T-cell abundance was found in *IDH*-mut gliomas. Gliomas bearing mutated *IDH* are characterized by abnormal production of the oncometabolite R-2-hydroxyglutarate (R-2-HG). R-2-HG impairs T-cell activity, CD8^+^ T-cell accumulation, and subsequent antitumor immunity (Kohanbash et al, 2017; Bunse et al, 2018). Therefore, MAP4K1-targeted therapy is suitable for *IDH-*wt and 1p/19q non-codeletion gliomas.

MAP4K1 increases the levels of IL-6R and IL-18R but decreases the cytokines IL-6, IL-7/IL-7R, and IL-18 in GBM cells. We suppose that there may be a negative feedback regulation mechanism of gene transcription through the IL-18R and IL-6R pathways. However, cytokines are secreted not only by cancer cells but also by other cell types in the tumor microenvironment. Previous studies have shown that multiple cytokines, including IL-6 and IL-10, activate their receptors on GBM cells to promote tumor progression (Jiang et al, 2017; Liu et al, 2021), whereas IL-7 reduces tumorigenicity and enhances the immune responses of CD8^+^ T cells (Aoki et al, 1992; Gunnarsson et al, 2010). IL-10 treatment significantly enhances cell growth and invasion of U87 GBM cells (Zhang et al, 2019). IL-6 stimulates cell growth and invasion of U87 and patient-derived primary GBM cells (Jiang et al, 2017). In this study, we explored the functional significance of the IL-18/IL-18R pathway in the proliferation and survival of GBM cells. These studies suggest that MAP4K1 remodels cytokine‒cytokine receptor networks (including IL-6/IL-6R, IL-7/IL-7R, and IL-18/IL-18R) to sustain GBM growth and progression in a cell-autonomous manner.

In addition to cytokine‒cytokine receptor pathways, we noticed that both the PI3K-AKT and MAPK pathways are involved in MAP4K1 downstream events. Aberration of the PI3K-AKT and MAPK pathways promotes GBM progression (Lin et al, 2017; Le Rhun et al, 2019). In this study, we found that MAP4K1 regulates the expression and membrane localization of IL-18R/IL-6R via the PI3K-AKT pathway. MLK3, the downstream kinase of the MAP4K1 pathway, predicts poor prognosis in patients with *IDH*-wt gliomas and regulates cytoskeleton remodeling of GBM cells by directly binding EPS8 (Zhu et al, 2020). MLK3-JNK (MAPK) signaling is related to EGFR activation-driven migration and invasion of GBM cells (Misek et al, 2017). Therefore, MAP4K1 inhibitors may cover a wide range of downstream targets to provide stronger antitumor responses than cytokine-targeted therapy.

In addition to the CD8^+^ T-cell-intrinsic exhausting pathway by MAP4K1, we provide additional evidence that GBM cell MAP4K1 inhibits CD8^+^ T-cell migration and infiltration. Our transcriptome analysis predicts that MAP4K1 may regulate chemokine CCL8 expression and secretion. We noticed that the levels of *CCL8* mRNA were positively correlated with those of *MAP4K1* in human gliomas and predicted poor clinical outcomes of patients (data not shown). Recent studies revealed that CCL8 recruits monocytes/macrophages into cervical cancer, postpartum breast cancer, or hepatocellular carcinoma (Chen et al, 2019; Farmaki et al, 2020; She et al, 2022). Tumor-associated macrophages impede CD8^+^ T cells from reaching cancer cells in solid tumors (Peranzoni et al, 2018). The number of infiltrated CD8^+^ T cells in gliomas is inversely correlated with glioma grades (Ghouzlani et al, 2021). Therefore, it is interesting to explore the involvement of CCL8 in MAP4K1-induced immune suppression.

Therefore, MAP4K1 is a reliable drug target for the treatment of malignant gliomas. Recently, MAP4K1 highly selective small-molecule inhibitors have been discovered, including GNE-1858, MK-12, A-745, and Gen-10 (Zhou et al, 2022). These small-molecule inhibitors are diverse in their activity and selectivity. Most of the inhibitors of MAP4K1 that are in development are in the preclinical research and discovery stage, which may bring new therapeutic prospects for gliomas.

In summary, our findings reveal that GBM cell MAP4K1 not only plays an oncogenic role in cell proliferation and tumor growth but also autonomously remodels the tumor immune microenvironment by resisting the trafficking of CD8^+^ TILs to tumor sites. Targeting the MAP4K1 pathway is a promising strategy for not only molecular-targeted therapy but also immunotherapy, which allows us to kill two birds with one stone for treating malignant gliomas.

## Materials and Methods

### Human glioma tissues

Glioma tissues of patients who had undergone surgery (WHO grade I, n = 6; grade II, n = 21; grade III, n = 22; and grade IV, n = 48) and para-tumor tissues (n = 26) were obtained from the Department of Pathology of the Affiliated Hospital of Xuzhou Medical University from 2016 to 2017. All samples were diagnosed by two pathologists according to the 2016 WHO classification criteria for central nervous system tumors. All glioma tissues and para-tumor tissues were made into a tissue microarray according to the standard procedure for the IHC assay.

Publicly available RNA-seq data of gliomas were collected from the Cancer RNA-Seq Nexus database (GSE59612), which included 19 normal brain tissues and 39 gliomas, and the CGGA database (http://www.cgga.org.cn) (Zhao et al, 2021), which includes two datasets, mRNAseq_325 (batch 2) and mRNAseq_693 (batch 1). Cases in the mRNAseq_325 (batch 2) and mRNAseq_693 (batch 1) datasets with complete clinical information, including histological grades, *IDH* wt or mut, 1p/19q codeletion or noncodeletion, and survival time, were selected for further analysis except for the correlation analyses of MAP4K1 with IL-18R and IL-6R. The dataset derived from the TCGA database (https://www.cancer.gov/ccg/research/genome-sequencing/tcga), which includes 166 GBM transcriptome sequencing datasets, was further analyzed by bioinformatics methods. The gene expression profile was measured using the Illumina HiSeq 2000 RNA Sequencing platform by the University of North Carolina TCGA genome characterization center. Gene set enrichment analysis was performed using the Bioconductor package clusterProfiler based on KEGG pathways.

### IHC assay

Human glioma tissue sections were deparaffinized with xylene and rehydrated with ethanol. Antigen retrieval was performed in high pressure with citrate for 3 min. The sections were blocked with 10% normal goat serum for 30 min at RT. Rabbit polyclonal anti-MAP4K1 antibody (1:200, ABS 159; Millipore) was incubated overnight at 4°C. Then, biotinylated goat anti-rabbit secondary antibody was incubated for 1 h at 37°C, followed by an avidin/biotin/peroxidase complex (Vectastain Elite ABC Kit #PK-6100; Vector Laboratories). Finally, the sections were stained using 3,3′-DAB, (ZLI9017; ZSGB Bio), and the nuclei were counterstained with hematoxylin. The Ki67-positive rates of human glioma tissues detected by IHC were acquired from the clinical pathological tests in the Pathology Department of the Affiliated Hospital of Xuzhou Medical University.

IHC detection of IL-18R (1:100, MAB840; R&D Systems), IL-6R (1:100, 227-SR; R&D Systems), and Ki67 (working solution, ZM-0166; ZSGB-BIO) in mouse cutaneous glioma tissues was performed by the two-step method. The procedures before primary antibody incubation were similar to the detection of MAP4K1 in human glioma tissues. After washing with PBS, horseradish peroxidase-conjugated goat anti-mouse/rabbit secondary antibody (working solution, PV6000; ZSGB Bio) was incubated for 1 h at 37°C. Then, DAB staining was carried out, and the nuclei were counterstained with hematoxylin. IHC staining images were acquired with an Olympus microscope.

### Evaluation of immunostaining tissues of human glioma samples

All samples of human glioma tissues were anonymized. The MAP4K1 IHC staining scores were evaluated blindly by two trained pathologists simultaneously by using a multiple-viewing microscope. The dominant staining intensity was scored as 0–3 (0, negative; 1, weak; 2, moderate; and 3, strong). The percentage of positive cells was scored into four different levels: 1 (0–25%), 2 (26–50%), 3 (51–75%), and 4 (76–100%). The levels of staining were calculated by multiplying the scores of staining intensity and percentage of positive cells, which were defined as immunoreactivity scores. According to the immunoreactivity scores, protein levels in gliomas and para-tumor tissues were defined into two levels: low level (0–4) and high level (6–12).

### Cell culture

GBM cell lines (human GBM cell lines U87, U118, T98G, and U251; normal human astrocyte NHAs; patient-derived GBM cells G1, G2, and G3; and the mouse GBM cell line GL261) were cultured in high sucrose DMEM, (#12000-014; Gibco) supplemented with 10% FBS, (Life Technologies) in an incubator containing 5% CO_2_ at 37°C. All human established cell lines were identified by short tandem repeat DNA fingerprinting from Cell Bank/Stem Cell Bank, the Committee of Type Culture Collection of Chinese Academy of Sciences (Shanghai, China) in August 2018.

#### Stable MAP4K1-KD cell line generation

Three non-overlapping shRNAs directed against human MAP4K1 and nontargeting shRNA in the human genome were obtained from GeneChem Company. The transduction MOI of lentivirus with human MAP4K1 shRNA was 10 in U87 and 20 in T98G cells. The KD efficiency was evaluated 96 h after lentivirus infection by immunoblotting. The most efficient sequence of shRNA-targeting human MAP4K1 was 5′-GCAAGGAAGAACATGGTTT-3′. The scramble sequence was 5′-TTCTCCGAACGTGTCACGT-3′. Mouse MAP4K1-targeting shRNA was obtained from GenePharma Company. The transduction MOI of lentivirus with mouse MAP4K1 shRNA was 20 in GL261 cells. The most efficient mouse MAP4K1 shRNA sequence was 5′-GCAUUCAGAGAAGAAGAUATT-3′; the negative control was 5′-UUCUCCGAACGUGUCACGUTT-3′. The MAP4K1 stable KD cell line and negative control cell line were created following the manufacturer’s protocol and were screened by puromycin.

#### siRNA transduction

The human MAP4K1 siRNA and the negative control siRNA were obtained from RiboBio. G1 cells were plated in six-well plates and cultured overnight. siRNAs were transduced by Lipofectamine 3000 (L3000001; Invitrogen) with a final concentration of siRNA of 50 nM. The MAP4K1 siRNA sequence was 5′-CCACCAAGATGCTCAGTCA -3′.

### *MAP4K1* gene knockout by the CRISPR/Cas9 system

The pSpCas9-2A-GFP (PX458) vector and two gRNAs were used to specifically knock out the human *MAP4K1* gene. The gRNA1 sequence was 5′-GCACGCCAACATCGTGGCCT-3′, and the gRNA2 sequence was 5′-TCGGCGGATCAGATCCACCC-3′, targeting exons 3 and 13, respectively. The gRNAs were annealed and inserted into the PX458 vector separately. PX458-MAP4K1 gRNA plasmids were cotransfected into T98G cells with Lipofectamine 3000 (Invitrogen), and monoclonal GFP-positive cells were screened by FCM and cultured in 96-well plates. Clones were identified by PCR, sequencing, and immunoblotting. The fragments of genomic DNA were amplified with two pairs of primers. The first-step PCR primers (which were designed outside of the spliced fragment) were forward primer F1 (5′-GATGCTGTGGAGGGACTCTGGC-3′) and reverse primer R1 (5′-CAGGTGCTGATGAGATTGTCTGG-3′). The second-step PCR primers (the upstream primer was designed outside of the spliced fragment, and the downstream primer was designed inside of the spliced fragment) were forward primer F1 (5′-TGGTGGCACTGAAGATGGTGAAGA-3′) and reverse primer R1 (5′-TCCAGCCTGGCAACAGAACAAGA-3′). Products of the first-step PCR were sequenced and confirmed by immunoblotting.

#### Rescue experiment in *MAP4K1* knockout T98G cells

*MAP4K1* cDNA was packaged and verified by GeneChem. The transduction MOI of *MAP4K1* lentivirus and the control virus was 20 in *MAP4K1* knockout T98G cells. A stable T98G cell line with MAP4K1 restoration was screened with 2 μg/ml puromycin and obtained after four cycles of screening.

### Tumorigenesis in glioma models

#### Animals and animal housing

Healthy female athymic nude mice aged 4–6 wk and C57BL/6 mice aged 6–8 wk were purchased from JUNKE Lab Animal Company. All procedures and experiments involving animals in this study were approved by the Institutional Animal Care and Use Committee and the Local Ethics Board. All the nude mice were kept and fed in a specific pathogen-free room at Xuzhou Medical University.

#### For subcutaneous tumor xenograft models

U87 or GL261 cells (5 × 10^6^ per mouse) stably transduced with human or mouse nontargeting shRNA lentivirus and MAP4K1 shRNA lentivirus were injected subcutaneously into the flanks of nude mice (eight mice per group) or C57BL/6 mice (11 mice per group, eventually 11 mice in the NC group and 10 mice in the KD group because one died in the process) in 150 μl of PBS. In the subcutaneous glioma model constructed by U87 cells, when palpable subcutaneous tumor xenografts formed, the size of the tumors was measured by Vernier calipers. The tumor size was measured every 2 d and evaluated using the following formula: V = ab^2^/2, where a and b (a > b) represented the tumor’s length and width. The subcutaneous tumor xenografts were then removed when the tumor size reached ∼1,500 mm^3^, weighed and photographed. In the subcutaneous glioma model constructed by GL261 cells, the mice were euthanized on the 28th d after injection. The tumor tissues and peripheral blood were collected for immune analyses.

#### For intracranial tumor xenograft models

U87 or GL261 cells (5 × 10^5^ per mouse) with nontargeting shRNA and MAP4K1 shRNA in 5 μl of PBS were stereotactically transplanted into the right cerebral cortex of nude mice (four mice per group, U87 cells) or C57BL/6 mice (four mice per group, GL261 cells) after anesthesia with 1.5% pentobarbital sodium. The microsyringe needle was positioned 2 mm to the right of bregma, 1 mm to the front of the coronal suture, and 3 mm below the surface of the skull using a stereotactic instrument. The intracranial glioma model constructed by U87 cells was euthanized on the 28th d after injection. The brain tissues were collected for hematoxylin and eosin (HE) staining to observe the growth of glioma. Mice in the intracranial glioma model constructed by GL261 cells were euthanized on the 20th d after injection. The brain tissues were collected for immunofluorescence detection of CD8^+^ T cells. All mice were monitored every day.

### HE staining

Brain tissues collected from the intracranial glioma model and the subcutaneous tumor tissues constructed by U87 cells embedded in paraffin were cut into 4 μm-thick slices. After xylene dewaxing and gradient alcohol dehydration, these slices were stained with hematoxylin for 2–5 min and rinsed with water. After differentiation with 1% hydrochloric acid for a few seconds and bluing for 1 min with lithium carbonate, the slices were washed with water. Then, slices were dyed with eosin for ∼30–60 s, followed by rehydration with gradient alcohol and transparentization with xylene. Slices were eventually sealed with neutral balsam. Staining images were acquired by an Olympus microscope.

### Cell viability assay

GBM cells (U87, 5 × 10^3^/well, T98G, 4 × 10^3^/well, G1, 5 × 10^3^/well) of different groups were seeded in 96-well plates in quintuplicate and were cultured for 0, 24, 48, 72 and 96 h. CCK8 (VC5001L; VICMED) was added, and the absorbance at 450 nm at each time point was measured by a microplate reader. The experiment was repeated in three independent experiments.

### EdU cell proliferation assay

The EdU assay was carried out by a BeyoClick EdU-594 cell proliferation kit (C0078S; Beyotime). Experimental and control cells (U87, 5 × 10^3^/well, T98G, 4 × 10^3^/well) were seeded into 96-well plates for 48 h, and the EdU assay was performed following the manufacturer’s instructions. Cells were incubated with EdU; (10 μM) for 2 h at 37°C and then fixed with 4% PFA for 15 min at RT. Then, the cells were treated with 0.3% Triton X-100 for 15 min at RT. Click Additive Solution was added and incubated for 30 min in the dark. Cell nuclei were stained with Hoechst 33342. The percentage of EdU-positive cells was calculated based on counts from 20 independent fields of view (100× magnification).

### Cell death and cell cycle evaluation

Cell death was assayed by an Annexin V-Alexa Fluor 647/PI assay kit (FMS AV647-100; FcMACS). GBM cells (U87, 2 × 10^5^/well, T98G, 1.5 × 10^5^/well, G1, 1.5 × 10^5^/well) from different groups were seeded in six-well plates. Cells were collected after 48 h. After washing two times with PBS, the cells were stained with Annexin V-Alexa Fluor 647 and PI for 15 min and analyzed by a flow cytometer (FACSCanto II). The collected data were analyzed by FlowJo software. The experiments were repeated in three independent experiments.

GBM cells starved in a serum-free medium for 36 h were seeded in six-well plates (U87, 2 × 10^5^/well, T98G, 1.5 × 10^5^/well, G1, 1.5 × 10^5^/well). A cell cycle distribution assay was performed after 72 h with a Cell Cycle Detection Kit (KGA512; KeyGEN BioTECH). Cells were collected and fixed with 70% icy ethanol overnight. The next day, the cells were washed twice with PBS and stained with PI for 1 h. Then, the samples were detected by FACS (Canto II), and the percentage of cells in each stage of the cycle was analyzed by MFLT32 software. The experiments were repeated in three independent experiments.

### Transcriptome analysis

Total RNA was extracted from MAP4K1-NC T98G and MAP4K1-KD T98G cells and quantified using a NanoDrop 2000c spectrophotometer (Thermo Fisher Scientific). Each group includes three biological duplications. Next-generation sequencing of the transcriptome was performed, and Gene Ontology (GO) enrichment and KEGG pathway enrichment were analyzed by Beijing Genomics Institution (BGI, China). A heatmap of DEGs in the cytokine‒cytokine receptor pathway was made by GraphPad Prism 7.0 software.

### Immunoblot analysis

Cells were collected, and Lorry’s method was used to determine the protein concentration. Equal protein samples were electrophoresed using 10% SDS‒PAGE gels. Cell total protein was separated and transferred onto nitrocellulose membranes. The membrane was blocked with 3% BSA in TBST buffer for 3 h at RT. Then, the primary antibody was added and incubated overnight at 4°C, followed by horseradish peroxidase-conjugated secondary antibody for 1 h at RT. Chemiluminescence reagent was used to detect the specific proteins. The band intensity was quantified by Quantity One software, and Adobe Photoshop was used to create the figures.

The following antibodies and dilutions were used: rabbit polyclonal anti-MAP4K1 (1:1,000, #4472; CST) for immunoblotting, mouse monoclonal anti-P-AKT (S473) (1:2,000, 66444-1-Ig; Proteintech), rabbit polyclonal anti-AKT (1:2,000, #AF6261; Affinity), mouse monoclonal anti-actin (1:10,000, 66009-1; Proteintech), mouse monoclonal anti-GAPDH (1:10,000, 60004-1; Proteintech), goat anti-mouse secondary antibody (1:10,000, SA00001-1; Proteintech), and goat anti-rabbit secondary antibody (1:10,000, SA00001-2; Proteintech).

### Real-time quantitative PCR (qRT-PCR)

Total RNA was extracted by TRIzol reagent (15596026; Invitrogen) according to the manufacturer’s instructions. cDNA was synthesized by reverse transcription from the total RNA with a reverse transcription reagent kit (R223-01; Vazyme Biotech). SYBR Green Master Mix (Q311-02; Vazyme Biotech) was used to make the reaction system for qRT-PCR. The reaction signals were collected with Applied Biosystems StepOnePlus. The primer sequences are listed as follows. IL-18R: forward primer, 5′-CCTTGACCCTTTGGGTGCTTA-3′; reverse primer 5′-CTCATGTGCAAGTGAACACGA-3′. IL-6R: forward primer, 5′-CCCCTCAGCAATGTTGTTTGT-3′, reverse primer, 5′-CTCCGGGACTGCTAACTGG-3′.

### Flow cytometry for receptor expression

Different groups of GBM cells (U87, T98G, G1) were collected. After washing with PBS twice, the cells were incubated with flow cytometry antibodies, including PE anti-human IL-18Rα (5 μl per 100 μl PBS, 313808; BioLegend) and/or PE-Cy7 anti-IL-6R (5 μl per 100 μl PBS, 352809; BioLegend), for 30 min at 4°C in the dark. After washing with PBS, the cells were analyzed with a flow cytometer. The results were analyzed by FlowJo software.

### Cytokine or neutralizing antibody treatment

*MAP4K1*^*+/+*^ and *MAP4K1*^*−/−*^ T98G cells were plated in 96-well plates and cultured in complete medium overnight. IL-18 (50, 100 ng/ml, 592102; BioLegend) was added to the complete medium. Cell viability was measured by CCK8 after 96 h. IL-18 (100 ng/ml) was added to the complete medium to detect cell viability at 24, 48, and 72 h. For the neutralization experiment, IL-18R neutralizing antibody (1, 5 μg/ml, MAB840; R&D Systems) was also added to complete medium, followed by cell viability detection after 96 h. IL-18 or IL-18R neutralizing antibody was replaced every 2 d.

### Immunofluorescence staining

Mouse brains collected from the intracranial glioma model were fixed in 4% PFA for 24 h, followed by soaking in 30% sucrose in PBS at 4°C for 48 h. The samples were cut into 20-μm-thick brain slices by a freezing slicer (Leica). Sections were blocked with 10% normal goat serum in PBS plus 0.3% Triton-100 (PBST) for 1 h. Then, the sections were permeabilized with Triton X-100 (0.2%, in PBS containing 10% normal goat serum) for 40 min at RT. Mouse anti-CD8 monoclonal antibody (1:300, sc-1177; Santa Cruz) was added and incubated overnight at 4°C. Then, Alexa Fluor 594 goat anti-mouse secondary antibody (A20185; Invitrogen) was incubated for 1 h at RT in the dark. The nuclei were stained with DAPI. Fluorescence images were obtained by a confocal laser-scanning microscope (Zeiss LSM-710; Oberkochen).

### Ex vivo analysis of T-cell infiltration by multicolor FACS

The subcutaneous glioma models constructed by GL261 cells in C57BL/6 mice were previously described for the glioma tumorigenesis model in vivo. Peripheral blood and tumor tissues of the GL261 subcutaneous glioma model were collected for immune regulation analysis. Subcutaneous tumor tissues were dissociated following the manufacturer’s protocol of a mouse tumor tissue dissociation kit (130-096-730; Miltenyi Biotech), and a single-cell suspension of tumor tissue was eventually obtained after filtration.

Moderate amounts of cells (1 × 10^6^) from the single-cell suspension were taken and stained with APC/Cyanine7 CD45 (0.25 μg/100 μl, 103115; BioLegend), PerCP/Cyanine5.5 anti-mouse CD3 (1 μg/100 μl, 100217; BioLegend), and PE/Cyanine7 anti-mouse CD4 (0.25 μg/100 μl, 100421; BioLegend) together for 30 min at 4°C in the dark for the analyses of CD4^+^ T-cell infiltration. Meanwhile, moderate amounts of tumor cells (1 × 10^6^) were taken from a single-cell suspension and stained with APC/Cyanine7 CD45, PerCP/Cyanine5.5 anti-mouse CD3 and APC anti-mouse CD8 (0.25 μg/100 μl, 100711; BioLegend) together for 30 min at 4°C in the dark for the analyses of CD8^+^ T-cell infiltration. Peripheral blood was divided in duplicate and stained with PerCP/Cyanine5.5 anti-mouse CD3 plus PE/Cyanine7 anti-mouse CD4 and PerCP/Cyanine5.5 anti-mouse CD3 plus APC anti-mouse CD8 directly for 30 min at 4°C in the dark. After erythrocyte lysis and washing, the stained cells were fixed with 1% PFA for FCM analyses by a flow cytometer (FACS CantoII). CD4^+^ and CD8^+^ T cells in tumor tissues were gated from CD45^+^ leukocytes.

### Conditioned media (CM)

GL261-NC and GL261-KD cells (5 × 10^5^ cells/well) were seeded in complete medium (DMEM including 10% FBS) in six-well plates. The next day, the cells were washed with PBS and then cultured in serum-free media for 24 h. Then, the media were collected, centrifuged to eliminate debris and was fresh for use in subsequent experiments.

### Chemotaxis assay

Chemotaxis of mouse lymph node-derived T cells was assessed using a 6.5 mm Corning Transwell cell culture chamber with a polycarbonate membrane (3.0 μm pore) following the manufacturer’s protocol. Cell suspensions of 5 × 10^5^ cells/0.1 ml in serum-free RPMI 1640 were loaded in the upper chamber compartment. CM from GL261-NC and GL261-KD cells was loaded in the lower chamber. Migrated cells were counted using a hemocytometer.

### Statistical analysis

All data are presented as the mean ± SD. All statistical analyses were performed using SPSS 16.0. The overall survival of glioma patients was assessed by the Kaplan‒Meier method. Correlation analysis was performed by Spearman correlation analysis and chi-square (*X*^2^) tests. Differences between groups were analyzed by unpaired *t* tests, Mann‒Whitney *U* tests and one-way ANOVA. The growth curve was analyzed by two-way ANOVA. *P* < 0.05 was considered statistically significant.

## Data Availability

All data are available from the corresponding author on reasonable request. The RNA-seq data from this publication have been deposited in Sequence Read Archive (SRA) database and assigned the identifier (accession code: PRJNA993003).

### Ethics statement

Human glioma tissue samples and patient derived glioma cells were approved for use in this study by the Ethics Committee of the Affiliated Hospital of Xuzhou Medical University (No. XYFY2018-KL056-01). Written informed consent has been provided by the participants’ legal guardian/next of kin before the surgery. All procedures and experiments involving animals in this study were approved by the Institutional Animal Care and Use Committee and the Local Ethics Board.

## Supplementary Material

Reviewer comments
