# Supplementary figures and images for: Glioblastoma cellular MAP4K1 facilitates tumor growth and disrupts T effector cell infiltration
Source: Life Sci Alliance. 2023 Sep 21;6(12):e202301966. doi: 10.26508/lsa.202301966 (PMC10514360; doi:10.26508/lsa.202301966)

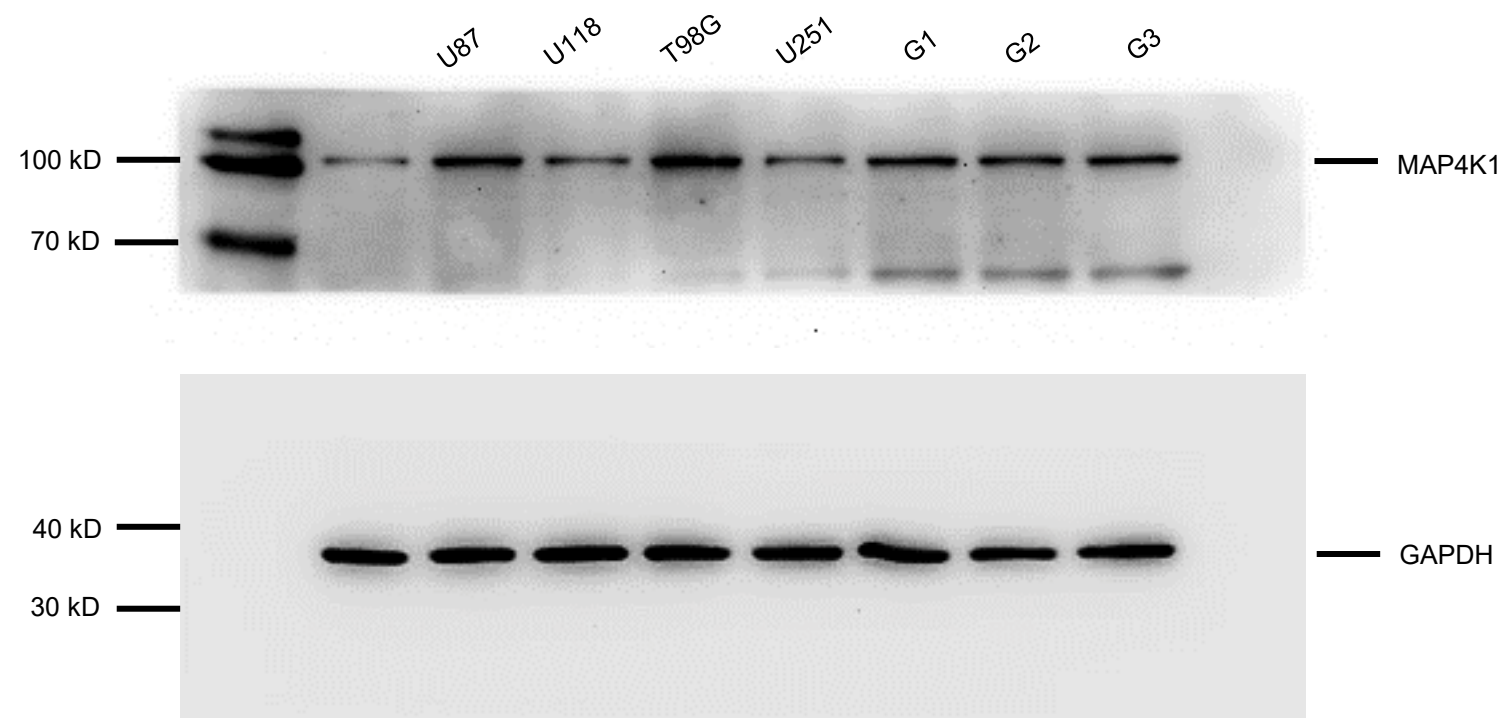

**Figure S1A**

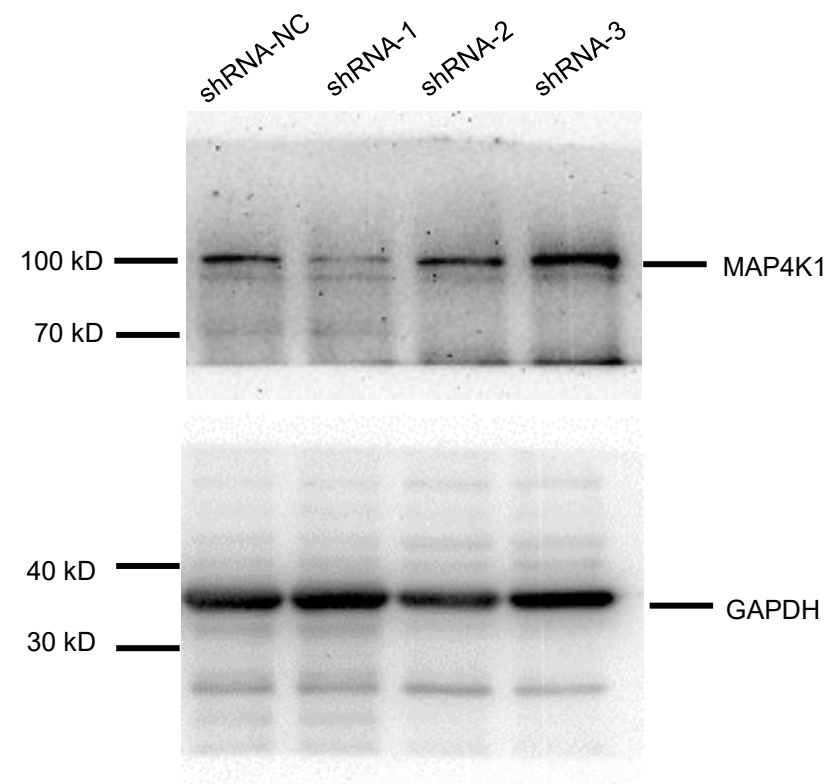

**Figure S1B**

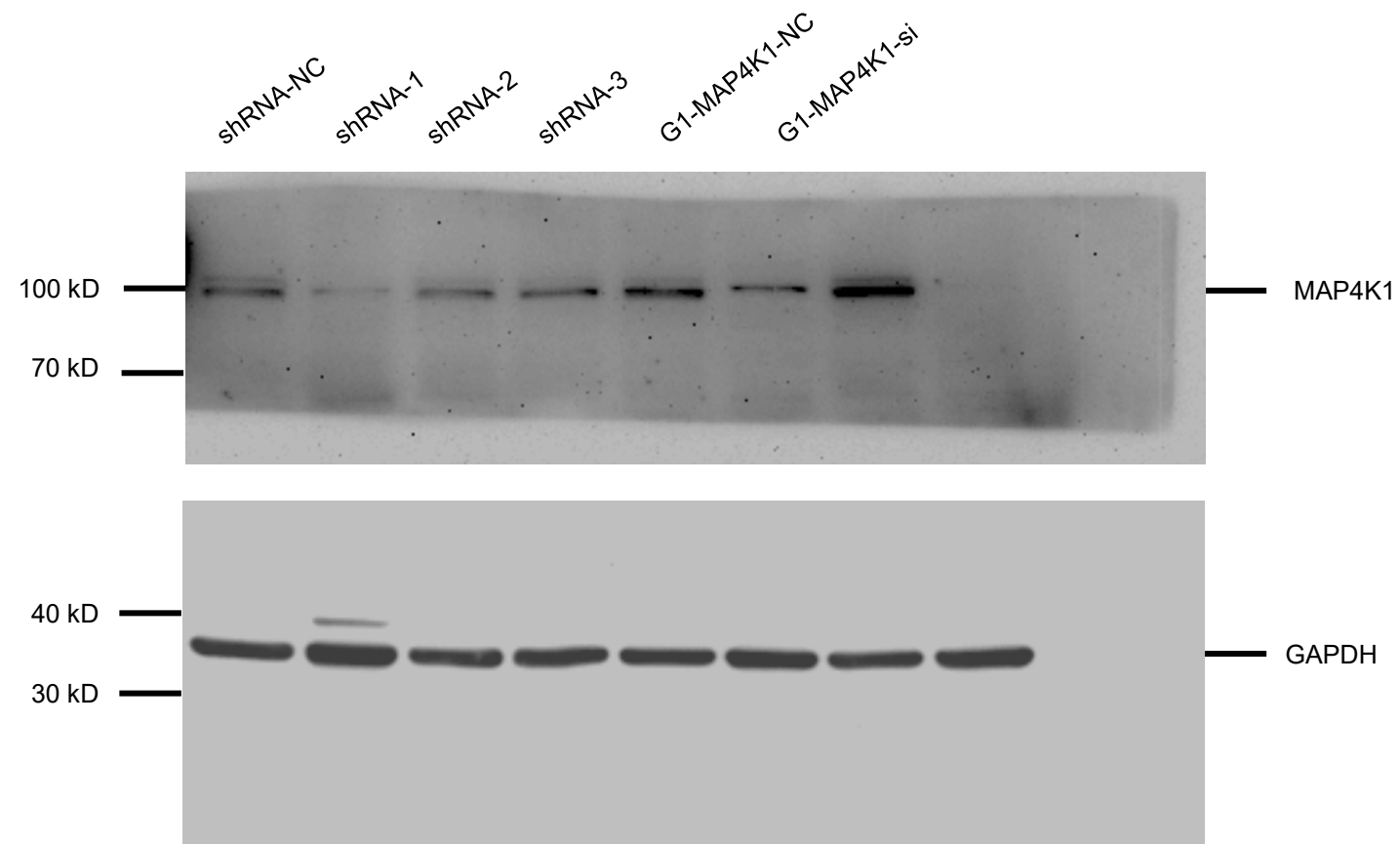

**Figure S1C, S2A**

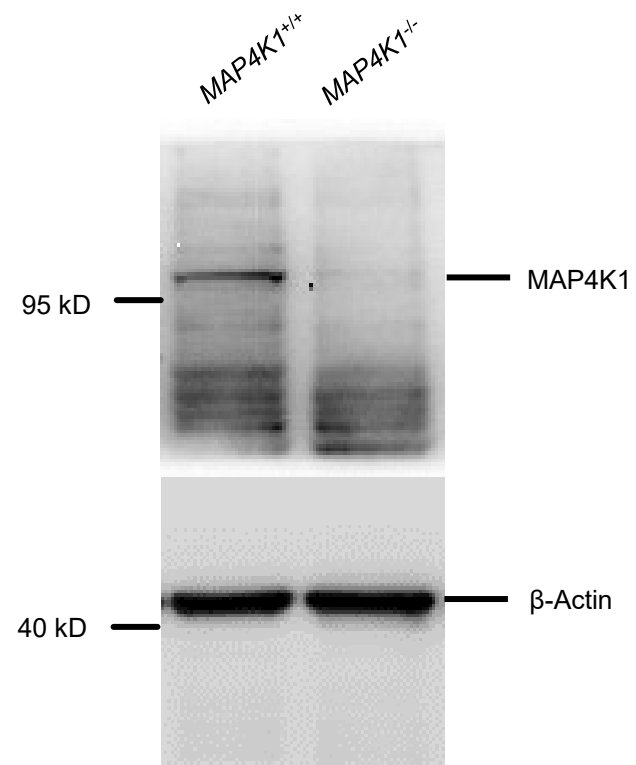

**Figure S1E**

Supplement: Supplementary file 1 [file LSA-2023-01966_SdataFS1_FS2.pdf]

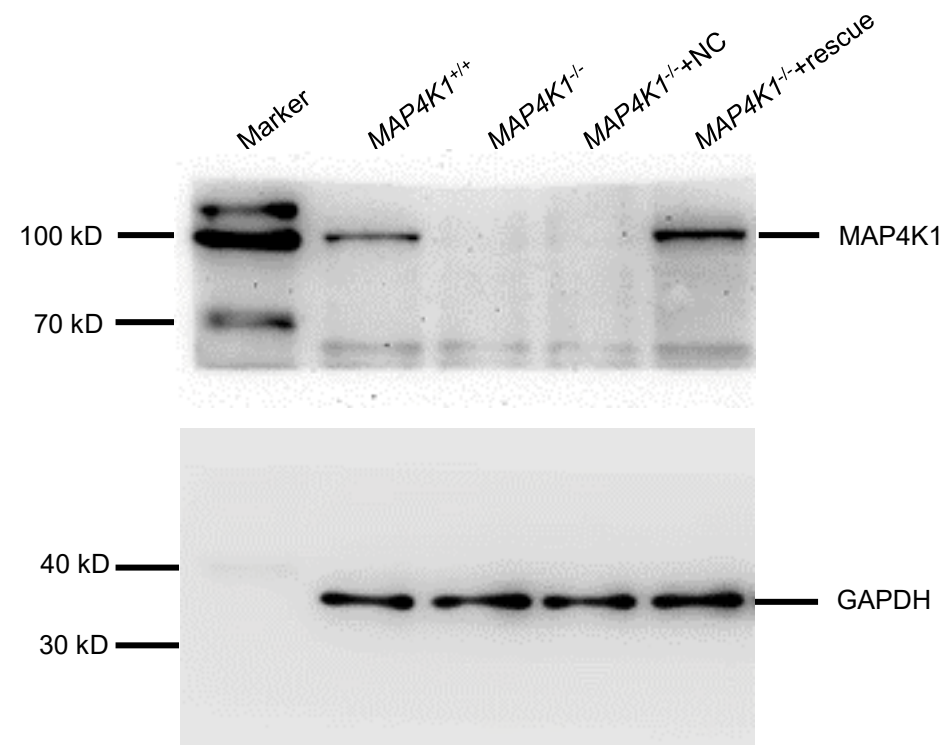

**Figure 8A**

Supplement: Supplementary file 2 [file LSA-2023-01966_SdataF8.pdf]

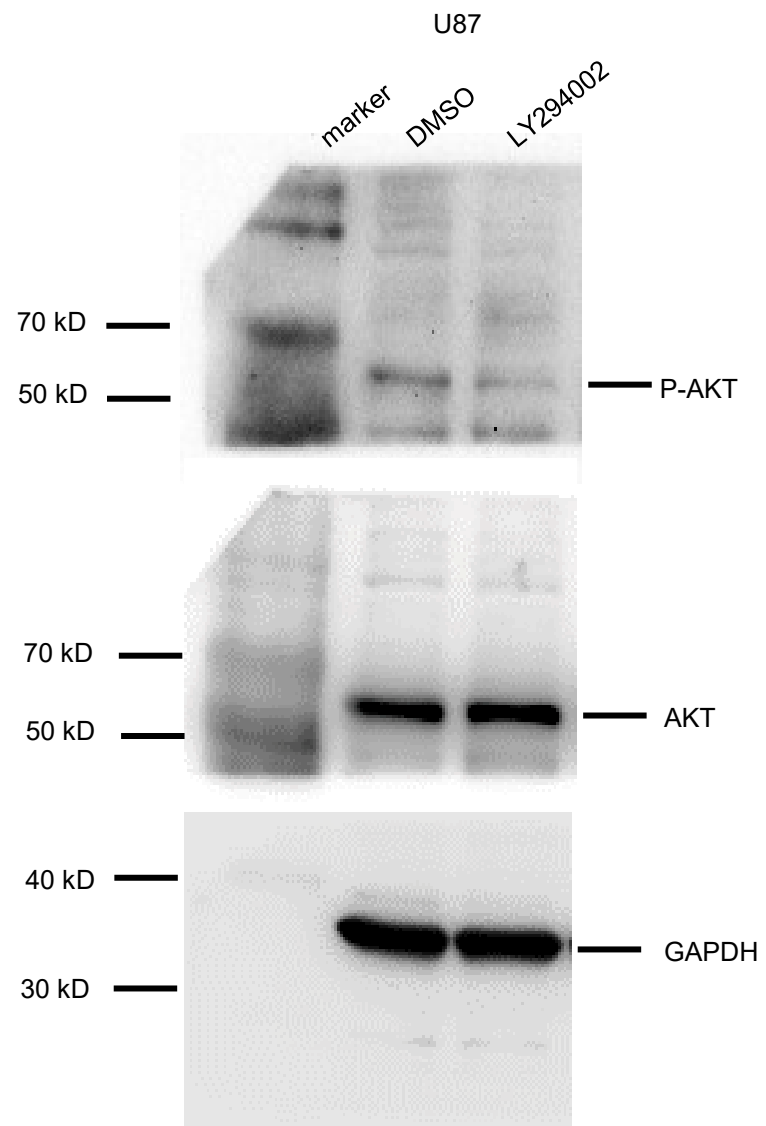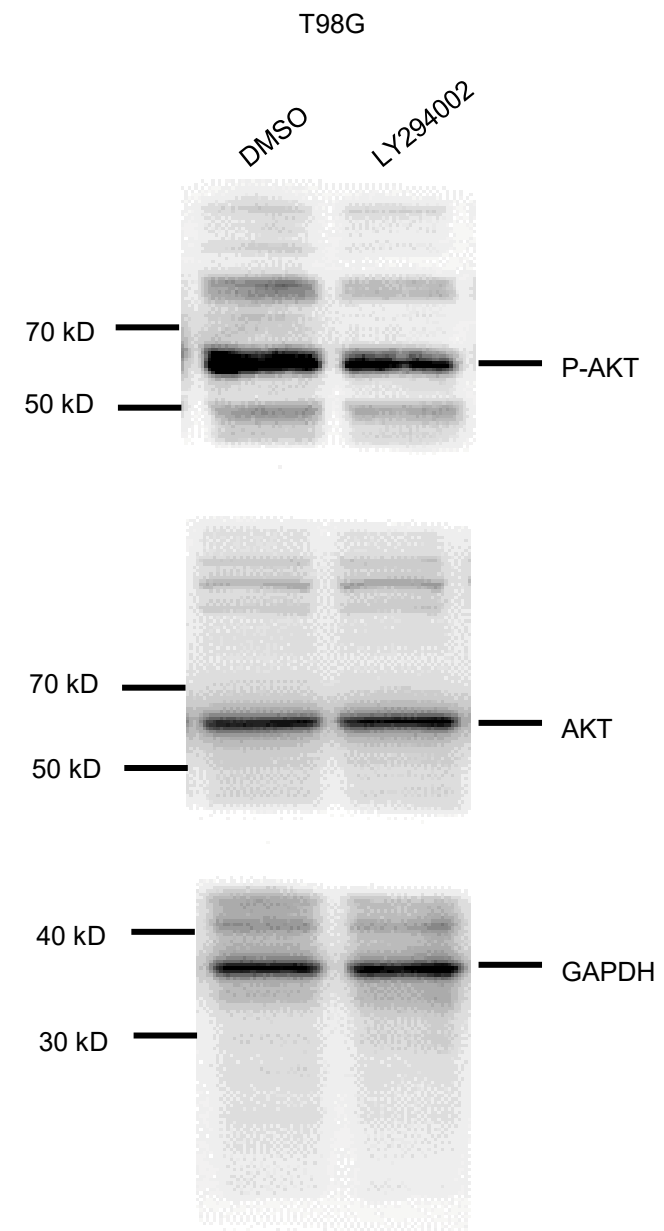

**Figure S5A**

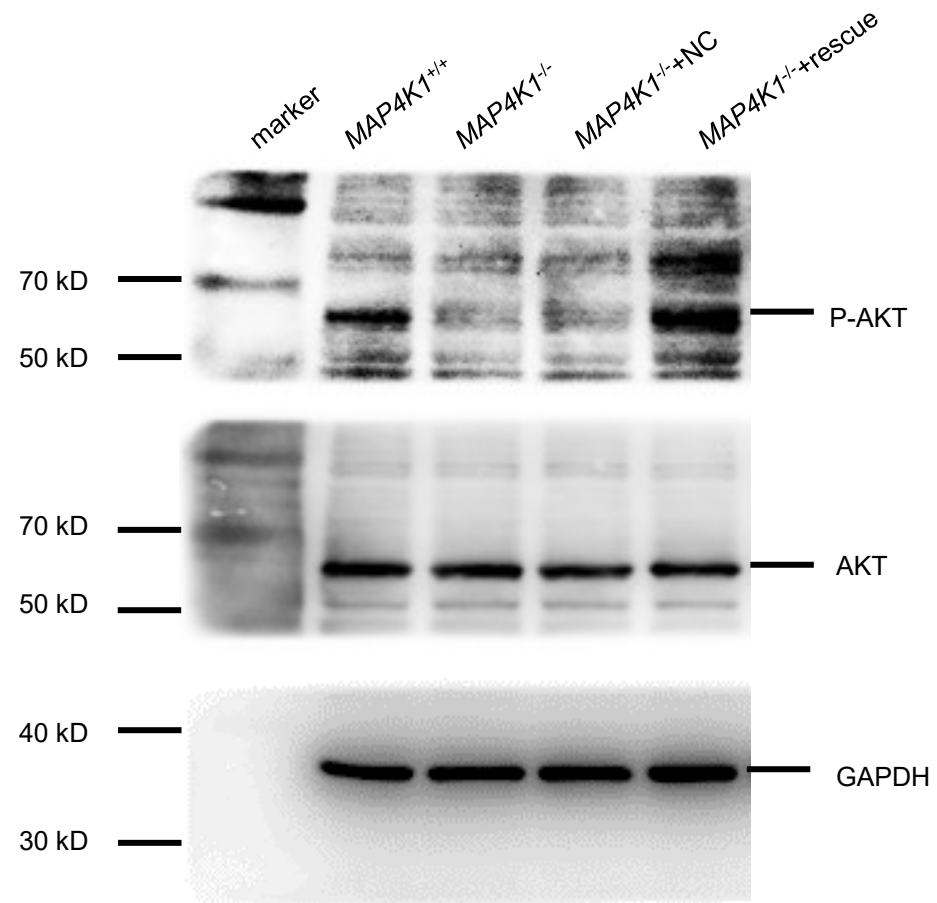

**Figure S5B**

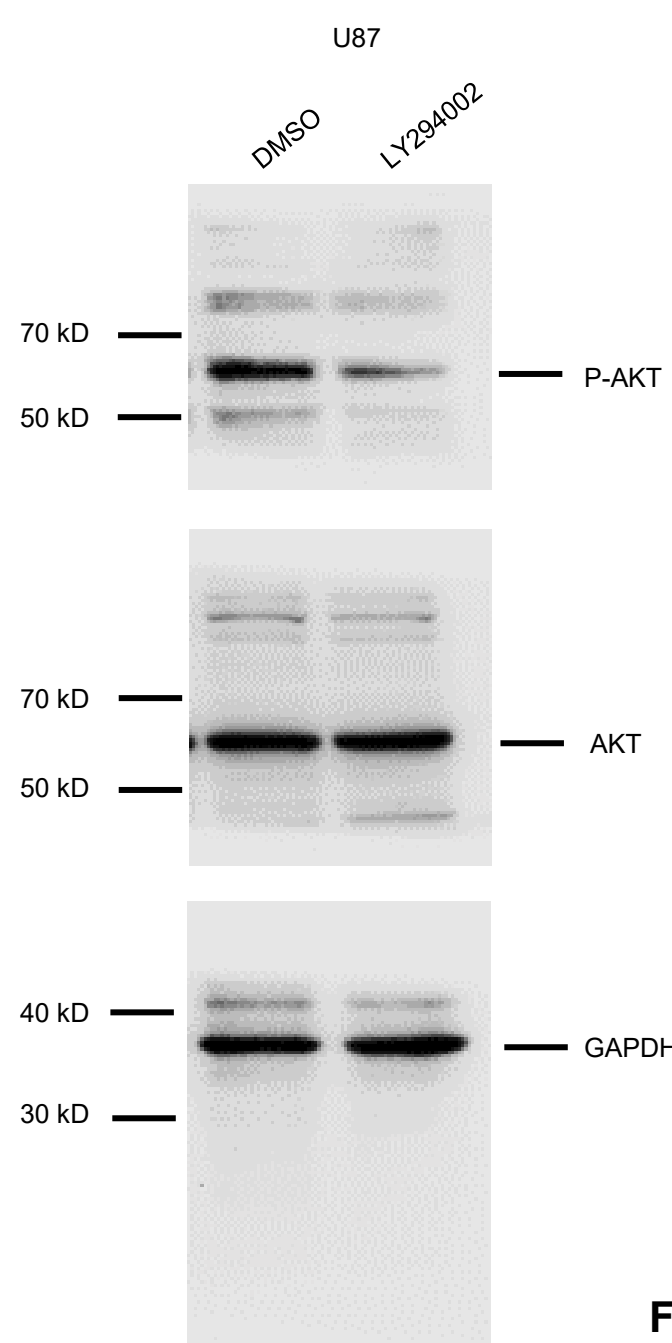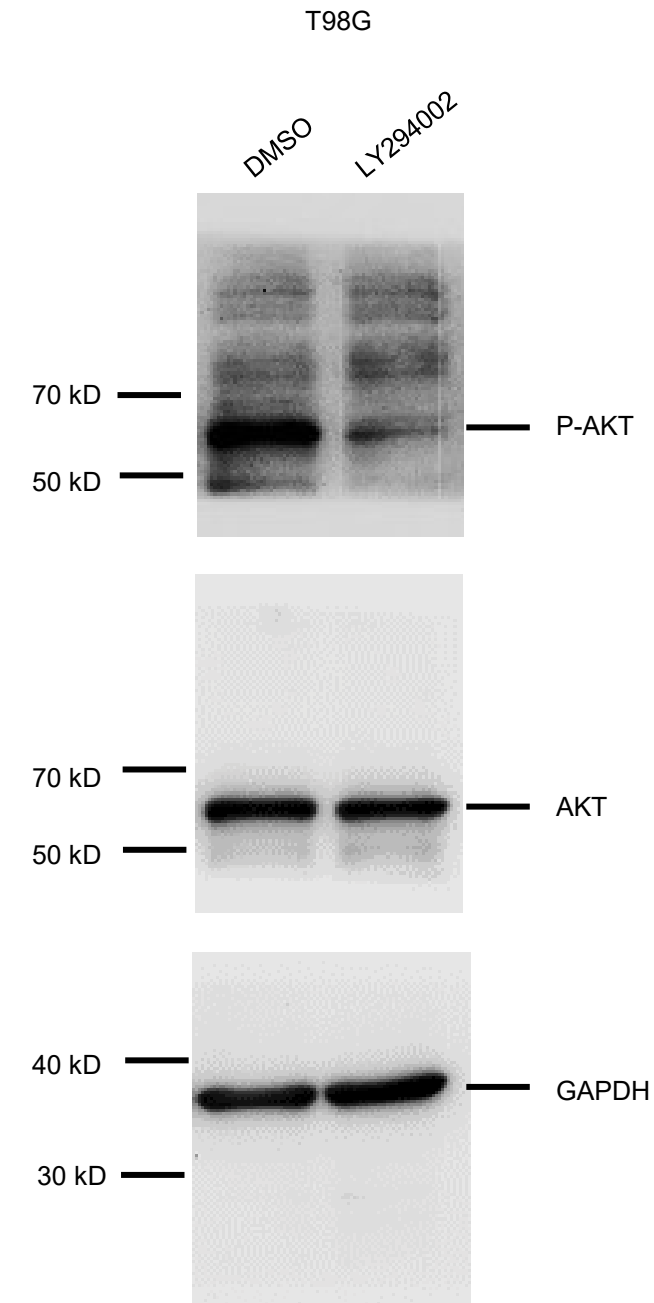

**Figure S5C**

Supplement: Supplementary file 3 [file LSA-2023-01966_SdataFS5.pdf]

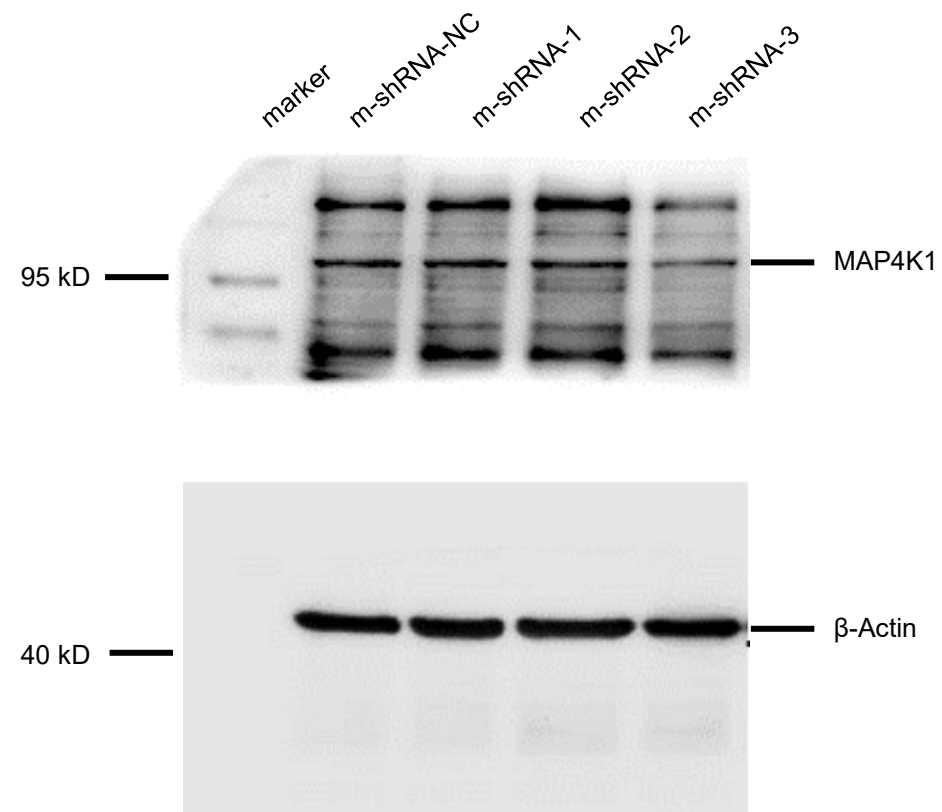

**Figure S6A**

Supplement: Supplementary file 4 [file LSA-2023-01966_SdataFS6.pdf]
